# Role of histone lactylation interference RNA m^6^A modification and immune microenvironment homeostasis in pulmonary arterial hypertension

**DOI:** 10.3389/fcell.2023.1268646

**Published:** 2023-09-12

**Authors:** Shuai-shuai Zhao, Jinlong Liu, Qi-cai Wu, Xue-liang Zhou

**Affiliations:** ^1^ Department of Cardiac Surgery, The First Affiliated Hospital, Nanchang University, Nanchang, China; ^2^ Institute of Translational Medicine, Shanghai University, Shanghai, China

**Keywords:** pulmonary arterial hypertension, histone lactylation, epigenetic modifications, post-translational modification, m^6^A, immune microenvironment

## Abstract

Pulmonary arterial hypertension (PAH) is a severe disease resulting from progressive increases in pulmonary vascular resistance and pulmonary vascular remodeling, ultimately leading to right ventricular failure and even death. Hypoxia, inflammation, immune reactions, and epigenetic modifications all play significant contributory roles in the mechanism of PAH. Increasingly, epigenetic changes and their modifying factors involved in reprogramming through regulation of methylation or the immune microenvironment have been identified. Among them, histone lactylation is a new post-translational modification (PTM), which provides a novel visual angle on the functional mechanism of lactate and provides a promising diagnosis and treatment method for PAH. This review detailed introduces the function of lactate as an important molecule in PAH, and the effects of lactylation on N6-methyladenosine (m^6^A) and immune cells. It provides a new perspective to further explore the development of lactate regulation of pulmonary hypertension through histone lactylation modification.

## 1 Introduction

PAH is a serious disease that involves pulmonary vasoconstriction, pulmonary vascular multiplication, and the development of plexiform lesions. At first, the right ventricle (RV) improves circulation by increasing contractility and ventricular wall thickness. With the progress of the disease, the RV gradually expands, eventually leading to right heart failure and even death (Harbaum et al., 2022). At the same time, PAH is also an important global health problem that can affect any age group. The prevalence of PAH is approximately 25 cases per population of 1 million (Maron et al., 2021). In the UK, the prevalence of PAH was 97 per million, with a female: male ratio of 1.8:1 (Galie et al., 2016), and in the United States, there are approximately 10.6 cases per 1 million adults (Badesch et al., 2010), with different epidemiological data of different types of PAH. With the development of medicine and the continuous efforts of doctors, the 5-year survival rate has increased from 34% to more than 60% through targeted treatment of PAH (Boucly et al., 2021). Even though currently available therapies focus on improving PAH symptoms and reducing pulmonary vasoconstriction, the mortality rate remains unacceptably high. Therefore, the identification of new pathways responsible for pulmonary vascular remodeling as well as identifying novel therapeutic targets are crucial.

Epigenetics emerging research has brought about many novel discoveries in PAH. Previous research has already demonstrated that m^6^A is a ubiquitous and abundant transcriptional modification. Mechanically, m^6^A modification affects multiple functions of mRNA, including transport, degradation, and translation, thus participating in various pathophysiological processes. The imbalance of m^6^A will lead to the occurrence and development of tumors, inflammation, cardiovascular disease, and immune disease (Efremova et al., 2020). The dynamic regulation of m^6^A affects the expression level of specific genes involved in PAH. In addition, inflammation and immune disorders are also involved in pulmonary vascular remodeling, especially through the secretion of cytokines and metabolic reprogramming (Xu et al., 2021). The pathological specimens of PAH patients showed the accumulation of perivascular inflammatory cells, such as macrophages, lymphocytes, and mast cells (Jia et al., 2020).

The crosstalk between epigenetics and metabolism plays a key role in gene expression, cell differentiation, and proliferation (Vasconcelos et al., 2020). Lactate has been found to be a signaling molecule and a metabolism regulator, participate in intercellular signal transduction and immune reaction (Shime et al., 2008), and play a key role in epigenomic reprogramming (Bhagat et al., 2019). Under hypoxia, cells stimulate intracellular lactate production by inhibiting oxidative phosphorylation and enhancing glycolysis, thereby increasing histone lactylation and promoting metabolic reprogramming (Zhang et al., 2019). The increase or decrease of lactate concentration has been shown to affect cell differentiation and function through multiple pathways. The increasing understanding of lactate has promoted the development of new targets. However, it just begin research histone lactylation in PAH. This review describes the regulation of m^6^A and the immune microenvironment by histone lactylation, affecting the occurrence and development of PAH.

## 2 Pulmonary arterial hypertension

In 1975, WHO published the first standardized hemodynamic criterion for pulmonary hypertension (PH) (Maron et al., 2018). In the resting state at sea level, check through the right heart catheterisation (RHC) technique, measure the mean pulmonary arterial pressure greater than 25 mmHg (mPAP ≥ 25 mmHg) (Al-Omary et al., 2020), and this definition has been followed ever since then. Until to 2018, the 6th World Symposium on Pulmonary Hypertension (WSPH) suggest that the diagnostic criteria for PH be modified to mPAP >20 mmHg, a pulmonary artery wedge pressure of 15 mmHg or lower, and a pulmonary vascular resistance of 3 Wood units or greater (Simonneau et al., 2019).

As shown in Table 1, PH is clinically divided into five major categories (Simonneau et al., 2019). The pathogenesis of PAH is complex and involves various factors, including vasoactive molecules (ET-1, Ang, PG, NO, etc.), ion channels (K^+^ channel, Ca^2+^ channel, and new cation channels), signaling pathways (MAPK pathway, PI3K/AKT pathway, Notch pathway, etc.) (Shafiq et al., 2021; Zhang et al., 2022), apoptosis resistance, oxidative stress, inflammation, and immune dysregulation (Norton et al., 2020). The pathological changes of PH include proliferation of pulmonary arterial endothelial cells (PAECs) along with the inflammatory response, proliferation of pulmonary arterial smooth muscle cells (PASMCs) and sustained contraction, and fibrosis of the external membrane and matrix remodeling (Rhodes et al., 2019). The main pathological feature of PH is pulmonary vascular remodeling caused by phenotypic changes in endothelial cells and muscularization of the vessel wall (Hautefort et al., 2019). This review focuses on elucidating the molecular mechanisms underlying the first type of epigenetic modifications of PH.

**TABLE 1:** Updated clinical classification of pulmonary hypertension (PH).

1. PAH	2. PH due to left heart disease	4. PH due to pulmonary artery obstructions
1. 1 Idiopathic PAH	2. 1 PH due to HF with preserved LVEF	4. 1 Chronic thromboembolic PH
1.2 Heritable PAH	2.2 PH due to HF with reduced LVEF	4.2 Other pulmonary artery obstruction
1.3 Drug- and toxin-induced PAH	2.3 Valvular heart disease	
1.4 PAH associated with:connective tissue disease, HIV infection, portal hypertension, congenital heart disease,schistosomiasis	2.4 Congenital/ acquired cardiovascular conditions leading to post-capillary PH	
1.5 PAH long-term responders to calcium channel blockers	3. PH due to lung diseases and/or hypoxia	5. PH with unclear and/or multifactorial mechanisms
1.6 PAH with overt features ofvenous/ capillaries (PVOD/PCH) involvement	3. 1 Obstructive lung disease	5. 1 Haematological disorders
1.7 Persistent PH of the newborn syndrome	3.2 Restrictive lung disease	5.2 Systemic and metabolic disorders
	3.3 Other lung disease with mixed restrictive/ obstructive pattern	5.3 Others
	3.4 Hypoxia without lung disease	5.4 Complex congenital heart disease
	3.5 Developmental lung disorders	

PAH, pulmonary arterial hypertension; HF, heart failure; PVOD, pulmonary veno-occlusive disease; PCH, pulmonary capillary haemangiomatosis; LVEF, left ventricular ejection fraction.

Early symptoms of PAH are not specific and usually include fatigue and chest tightness. As the disease progresses, symptoms gradually become more severe, including dyspnea, syncope, chest pain and right heart failure. Experts believe that early diagnosis and treatment can improve survival (Simonneau et al., 2019). The treatment of PAH includes general treatment, special drug treatment, surgical treatment, and targeted drug therapy. General treatment includes: activity and rehabilitation, anticoagulant therapy, diuretic and cardiovascular active drug therapy, oxygen therapy, anemia improvement and iron supplementation therapy, and psychosocial support. The specific drug treatments include: calcium channel blockers (CCB); endothelin receptor antagonists (ERA) consisting of bosentan, ambrisentan, and macitentan; 5-phosphodiesterase inhibitor (sildenafil, tadalafil); guanylate cyclase agonist (sGC) include Adempas; prostacyclin analog (epoprostenol, treprostinil, iloprost) and prostacyclin receptor agonist (selexipag) (Humbert et al., 2022).

Additionally, combination therapy is considered a standard treatment method in PAH. In spite of the fact that these treatments can improve the life quality and survival of patients, they do not cure the disease, the long-term prognosis is poor and the mortality rate is high. Therefore, the development of new drugs and the search for new treatments are the key to the treatment of PAH.

## 3 Mechanism of m^6^A methylation-modified mRNA affecting the development of PAH

### 3.1 The structure and function of m^6^A

A large number of research have shown that epigenetic modifications play an important role in regulating cell proliferation, protein synthesis, and gene transcription, including methylation, histone lactylation modification, and microRNA dysregulation. It is worth noting that m^6^A is a key regulator of mRNA stability, protein expression, and other cellular processes (Ries et al., 2019). The m^6^A peaks are mainly found in the open reading frame (ORF) (Li et al., 2018), the 3′-untranslated regions (UTRs), and near the stop codons of the mRNA (Ke et al., 2015). Mechanistically, m^6^A affects all stages of RNA metabolism, including translation, stabilization, and degradation, and plays a key role in the pathological and physiological processes of cells (H. Huang et al., 2019).

The mRNA methylation modifications are dynamically regulated by methyltransferases, demethylases, and methylation-binding proteins to maintain normal gene expression. Among them, the regulators involved are: methyltransferase including METTL3 (methyltransferase-like3) (Vu et al., 2017), METTL14 (methyltransferase-like14) (Chen et al., 2020), METTL16 (methyltransferase-like16) (Pendleton et al., 2017), WTAP (Wilms tumor 1associated protein) (Zhu et al., 2020), RBM15 (RNA binding motif protein15) and zinc finger CCCH-type containing 13 (ZC3H13) (Wen et al., 2018). The demethylases FTO (FAT mass and obesity-associated protein) (Mathiyalagan et al., 2019) and ALKBH5 (ALKB homologue5 protein) (Zhang et al., 2017) both are the ALKB protein family, and belong to the ferric hydride/ketoglutarate-dependent dioxygenase. The m^6^A reader protein recognizes mRNA and binds to it to achieve corresponding functions. One class of direct and robust m^6^A readers are proteins containing the YT521-B homology (YTH) domain, the YTH domain of the m^6^A reader protein is composed of 134 amino acids (Zaccara, and Jaffrey, 2020), including YTH domain family 1–3 (YTHDF1-3) (Gao et al., 2019; Li et al., 2020) and YTH domain containing 1–2 (YTHDC1-2) (Roundtree et al., 2017; Jain et al., 2018) in humans, were confirmed to regulate the mRNA processing, translation, and degradation processes (Table 2). How to maintain the above molecular expression level in homeostasis is the key to preventing vascular dysplasia and elevated pulmonary arterial blood pressure.

**TABLE 2:** The structure and function of m^6^A.

Type	Regulator	Function	References
m^6^A writer	METTL3	catalyzes m^6^A modification	(Vu et al., 2017; Wang et al., 2020)
	METTL14	helps METTL3 to recognize the subtract	(Chen et al., 2020; Li et al., 2020)
	METTL16	catalyzes m^6^A modification	(Pendleton et al., 2017)
	WTAP	contributes to the localization of METTL3-METTL14 heterodimer to the nuclear speckle	(Ping et al., 2014; Zhu et al., 2020)
	RBM15	binds the m^6^A complex and recruit it to special RNA site	(Patil et al., 2016)
	ZC3H13	bridges WTAP to the mRNA-binding factor Nito	(Wen et al., 2018)
m^6^A eraser	FTO	removes m^6^A modification	(Mathiyalagan et al., 2019; Mauer et al., 2017; Su et al., 2018)
	ALKBH5	removes m^6^A modification	(Guo et al., 2020; Tang et al., 2018; Zhang et al., 2017)
m^6^A reader	YTHDF1	enhances mRNA translation	(Gao et al., 2019; Shi et al., 2018; Wang et al., 2015
	YTHDF2	promotes mRNA degradation	(Du et al., 2016; Li et al., 2020)
	YTHDF3	enhances translation and degradation by interacting with YTHDF1 and YTHDF2	(Gao et al., 2019; Shi et al., 2017)
	YTHDC1	contributes to RNA splicing and export	(Roundtree et al., 2017; Zhu et al., 2021b)
	YTHDC2	enhances the translation of target RNA and reduces the abundance of target RNA	(Jain et al., 2018)
	HNRNPC	mediates mRNA splicing	(Wu et al., 2018)

Immunofluorescence showed that METTL3 is located on the nuclear spots rich in mRNA splicing factors and has a potential regulatory role in mRNA metabolism (Vu et al., 2017). Previous research showed that METTL3 might promote the development of thyroid cancer through the methylation modification of TCF1 (Wang et al., 2020). In mammals, both METTL3 and METTL14 are highly conserved, and both form stable heterodimers. Among them, METTL4 is an snRNA m^6^Am methyltransferase involved in the regulation of pre-mRNA splicing (Chen et al., 2020). Li et al. found that METTL14 may contribute to hepatocellular carcinoma progression through modulation of m^6^A methylation of cysteine sulfinic acid decarboxylase, glutamic-oxaloacetic transaminase 2, and cytokine signaling suppressor 2 (Li et al., 2020). The methyltransferase WTAP interacts with METTL3 and METTL14 to jointly regulate the m^6^A levels of mRNA transcription (Ping et al., 2014). METTL16, a homolog of METTL3, regulates the expression of human MAT2A, controls cellular SAM levels, and is also a methyltransferase of U6 snRNA (Pendleton et al., 2017). In addition, a study has shown that at least 78 m^6^A residues of XIST are highly methylated in human cells. Among them, RBM15 and RBM15B mediate the methylation of adenosine nucleotides in the common motif of m^6^A in XIST and mRNA (Patil et al., 2016). The above methyltransferases achieve different functions by modifying different stages of mRNA.

The demethylases FTO and ALKBH5 play powerful functions in RNA translation, processing, and splicing (Tang et al., 2018). In terms of modified bases, the m^6^Am is one of the most common near the first coding nucleotide of the 7-methylguanosine cap of mRNA. FTO preferentially demethylates m^6^Am and reduces the stability of mRNA (Mauer et al., 2017). The regulation of mRNA function by FTO leads to FTO-dependent changes in m^6^A demethylated protein levels (Su et al., 2018). A study found that FTO plays a key role in cardiac remodeling. Compared with healthy heart tissue, m^6^A modification was increased and FTO expression was significantly decreased in heart failure and myocardial infarction regions (Mathiyalagan et al., 2019). ALKBH5 is the second discovered m^6^A demethylase, which is similar to the m^6^A demethylation activity of FTO (Zhang et al., 2017). A study showed that ALKBH5 overexpression can inhibit the proliferation of pancreatic cancer cells *in vitro*, whereas ALKBH5 knockdown promoted the progression of pancreatic cancer (Guo et al., 2020) (Table 2). This suggests that m^6^A demethylase achieves distinct cellular functions by interfering with mRNA stability.

The m^6^A binding protein YTHDF1 is translocated from the cytoplasm to the nucleus, where it initiates and enhances translation in a manner that is dependent on the eIF3 initiation factor (Wang et al., 2015). YTHDF1 gene deletion leads to decreased memory and learning, while YTHDF1 expression enhances memory and learning (Shi et al., 2018). Transporting mRNA targets to cytoplasmic processing bodies and promoting their degradation are the functions of YTHDF2. The CCR4-NOT deadenylase complex partially promotes the degradation of target transcripts by cytoplasmic YTHDF2 (Du et al., 2016). The YTHDF3 protein interacts with the YTHDF1 and YTHDF2 proteins to enhance translation and degradation (Shi et al., 2017). A structural and binding study indicates that the YTH domain of YTHDC1, one of the core members of the YTH family proteins, preferentially recognizes the GG (m^6^A)C sequence (Roundtree et al., 2017). It has been shown that YTHDC1 promotes the proliferation of cancer cells, the formation of tumors and the migration of cells (Zhu et al., 2021). In addition, YTHDC2 binds to the consensus motif of m^6^A more preferentially than other members of the YTH family, improving translation efficiency and reducing mRNA bundling (Jain et al., 2018). Heterogeneous nuclear ribonucleoproteins (HNRNPs) regulate alternative splicing or processing of target transcripts, including HNRNPC, HNRNPG, and HNRNPA2B1 (Wu et al., 2018) (Table 2).

### 3.2 m^6^A methylation-modified mRNA affects the occurrence and development of PAH

The physiological function of m^6^ A in the cell is mediated by different mechanisms, m^6^ A regulates the stem cell fate by modifying mRNA (Li et al., 2018). In the past 2 years, many studies have reported that the occurrence and development of PAH is closely associated with epigenetic modification of mRNA, particularly m^6^A methylation modification (Zhu et al., 2021). Zeng et al. had confirmed that increased m^6^A methylation in PAH (Zeng et al., 2021). In addition, some studies have demonstrated that METTL3 (Qin et al., 2021), METTL14 (Zhou et al., 2021), YTHDF1 (Hu et al., 2021), and YTHDF2 (Qin et al., 2021) are involved in PASMC proliferation and pulmonary vascular remodeling.

METTL3 plays an important role in the pathogenesis of hypoxia-induced PAH. Qin et al. pointed out that METTL3 is abnormally overexpressed in PASMCs of PAH. However, downregulation of METTL3 inhibited hypoxia-induced proliferation and migration of PASMCs (Qin et al., 2021). Meanwhile, study revealed that YTHDF2 regulates RNA metabolism by localizing bound mRNAs to degradation sites (Fei et al., 2020). There was a significant upregulation of YTHDF2 in PASMCs under hypoxia. Since YTHDF2 recognizes m^6^A on PTEN mRNA, METTL3 decreases the stability of PTEN mRNA and accelerates its degradation via YTHDF2. The PI3K/Akt signaling pathway is activated in response to the reduced PTEN level, further promoting the proliferation of PASMCs (Qin et al., 2021). In addition, research also shows that SETD2 catalyzes H3K36me3 and plays a key role in hypoxic PAH formation (Yao et al., 2020). Hypoxia-induced PAH mice showed increased expression of SETD2 and m^6^A transcript METTL14 in PASMCs, and SETD2-specific knockout in SMC ameliorated PAH and also decreased METTL14. This suggests that hypoxia-induced PAH is caused by METTL14-mediated m^6^A modification and SETD2-mediated H3K36me3 modification (Zhou et al., 2021) (Table 3). Thus, the occurrence and development of PAH are commonly promoted by multiple m6A methylation modifications.

**TABLE 3:** Role of m^6^A methylation modification in PAH.

Type	Regulator	Expression	Mechanisms	References
m^6^A writer	METTL3	Increase	METTL3/YTHDF2/PTEN axis promotes the hypoxia induced PAH.	(Qin et al., 2021; Zeng et al., 2021)
	METTL14	Increase	SEDT2/METTL14-mediated m^6^A methylation contributes to the hypoxia induced PAH in mice	(Zhou et al., 2021)
m^6^A reader	YTHDF1	Increase	YTHDF1 regulates the PAH through translational control of MAGED1	(Hu et al., 2021; Zeng et al., 2021)
	YTHDF2	Increase	METTL3/YTHDF2/PTEN axis promotes the hypoxia induced PAH.	(Qin et al., 2021)
	YTHDC1	Increase	FENDRR with YTHDC1 regulates PAH by mediating DRP1 DNA methylation	(Wang et al., 2022)
	HNRNPA2B1	Increase	Interfered with RNA splicing, transport, and maturation which mediate the phenotype translational of PASMCs	(Zheng et al., 2022)
m^6^A eraser	FTO	Decreased	—	(Zeng et al., 2021)
	ALKBH5	Decreased	—	(Zeng et al., 2021)

Recently, YTHDF1 has been shown to be overexpressed in human and rodent PAH samples and hypoxic PASMCs. The researchers found that MAGED1 regulates PAH pathogenesis by directly targeting m^6^A. YTHDF1 promoted PASMC proliferation and the development of PAH by increasing MAGED1 translation, and MAGED1 knockdown reduced hypoxia-induced proliferation of PASMCs by downregulating proliferating cell nuclear antigen (PCNA) (Hu et al., 2021). Meanwhile, Wang et al. showed that the expression of YTHDC1 was enriched in PAECs under hypoxic conditions and mediated FENDRR involved in the hypoxia-induced proliferation of PAECs (Wang et al., 2022). In addition, DEGs and HNRNPA2B1 target genes overlapped in PASMCs, indicating that HNRNPA2B1 was upregulated in PASMCs. HNRNPA2B1 regulates the Wnt signaling pathway, cAMP signaling pathway, P53 signaling pathway, and cell cycle of muscle cell differentiation, and participates in the signaling pathway by modifying m^6^A modification (Zheng et al., 2022) (Table 3).

## 4 The immune microenvironment dysequilibrium promotes the development of PAH

Recent studies have found that the occurrence and development of PAH is the result of a variety of cell interactions, which is not only related to PAECs dysfunction, PASMCs phenotypic switching and fibroblast activation, moreover, it is also closely related to the immune microenvironment imbalance. Accumulating evidence suggests that inflammation is a major contributor to vascular remodeling in PAH (Xu et al., 2021). The disorder of the immune microenvironment plays an important role in the development of PAH, and the immune system regulates PAH via multiple mechanisms.

**FIGURE 1:**
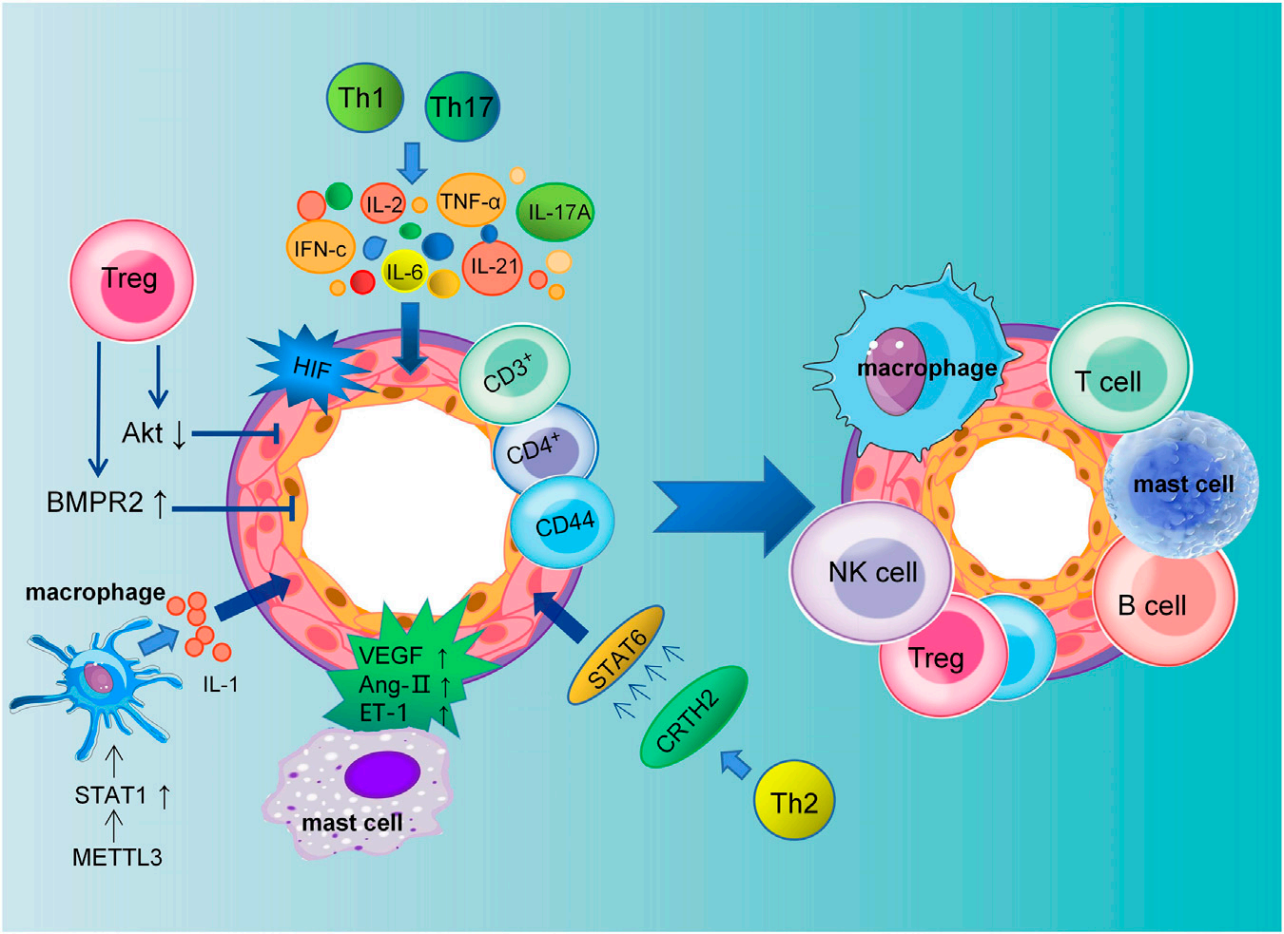
Schematic representation of pulmonary artery remodeling promoted by immune microenvironment dysregulation. The dysfunction of macrophages, mast cells, T cells, B cells, NK cells and Tregs together lead to pulmonary vascular remodeling in PAH. Th1 and Th17 cells mediate the inflammatory response in PAH by producing IL-2, IL-6, IL-21, IL-17A, IFN-c, and TNF-α. Meanwhile, PASMCs proliferation is promoted by CRTH2 from Th2 lymphocytes through the activation of STAT6. Tregs inhibit the proliferation of PASMCs by decreasing Akt activity and regulating the kinase of an extracellular signal. Tregs can reduce perivascular inflammation and PAECs apoptosis through upregulation of BMPR2. In addition, VEGF, Ang II, and ET-1 secreted by mast cells are all involved in the remodeling of the pulmonary vasculature.

Mechanistically, immune cells induce an inflammatory response by releasing various types of inflammatory mediators and cytokines to bind to cytokines receptors on vascular endothelial cells, smooth muscle cells, and fibroblasts (Guihaire et al., 2021; Tang et al., 2021). Pulmonary vascular and perivascular inflammation is one of the major factors leading to vasoconstriction and vascular remodeling. PAEC dysfunction leads to the release of vasoconstrictive and inflammatory factors that promote excessive proliferation of PASMCs and pulmonary artery constriction (Florentin et al., 2018). Extensive research has shown that different subsets of T lymphocytes play distinct roles in PAH, including helper T lymphocytes (Th cells), cytotoxic T lymphocytes, and regulatory T lymphocytes (Tregs). Among them, Th1 and Th17 cells are involved in the autoimmune and inflammatory response of PAH by producing IL-2, IL-6, IL-21, IFN-c and TNF-α (Steiner et al., 2009). Meanwhile, Maston et al. found that Th17 cells promote the progression of hypoxia-induced PAH in rats by releasing IL-17A (Maston et al., 2017) (Figure 1).

Elevated levels of cytokines and chemokines have been found in patients with idiopathic PAH (Perros et al., 2013). Meanwhile, The expression of CRTH2 (chemoattractant receptor homologous molecule expressed on Th2 cells) was increased in both circulating CD3^+^CD4^+^ T cells in idiopathic PAH patients and rodent models of PAH. Chen et al. have shown that CRTH2 promotes PASMC proliferation by activating STAT6 (Chen et al., 2018; Harbaum et al., 2016). In addition to regulating collagen synthesis and proliferation of PASMCs, CD44^+^ T cells play a key role in pulmonary vascular remodeling, immune regulation, and phenotypic transformation (Isobe et al., 2019). The above studies suggest that the release of inflammatory factors promotes the progression of PAH.

In humans and mice, studies have shown that Tregs make up approximately 5%–10% of peripheral blood lymphocytes (Elkord, 2009). They inhibit autoimmunity and maintain immune homeostasis. Previous studies have shown that abnormal Tregs may impair the anti-inflammatory function of PAECs and play a key role in the pathogenesis of PAH. A decreased number of Tregs was observed in the pulmonary vessels of PAH patients, while an increase was observed in the peripheral circulation, indicating the decreased suppressive function of Tregs (Huertas et al., 2016). In addition, Tregs are involved in the regulation of adaptive and innate immunity. In PAH, Treg deficiency promotes the emergence of destructive macrophage-based immunity that damages the endothelium and leads to vascular remodeling (Tian et al., 2013). In conclusion, normal function of Tregs may limit pulmonary vascular damage and prevent the development of PAH.

Bone morphogenetic protein receptor type 2 (BMPR2) is also involved in the pathogenesis of PAH, which is mainly secreted by PAECs and feeds back to them, then inhibits their proliferation and differentiation (Diebold et al., 2015). Research has shown that Tregs function by upregulating BMPR2 expression to decrease endothelial cell apoptosis and perivascular inflammation. However, as a consequence of decreased BMPR2 secretion in injured PAECs, they are much more susceptible to PAH (Hong et al., 2008). In the meantime, the study by Chu et al. found that Tregs inhibit PASMC proliferation and PAH development by inhibiting Akt and extracellular signal-regulated kinase (Chu et al., 2015). According to several studies, macrophages are involved in the progression of PAH through their inflammatory response (Zhang et al., 2020). In addition, accumulation of B cells and macrophages after 1 week in Treg-deficient rats exposed to SU5416 (Tamosiuniene et al., 2011). Jia et al. have shown that by reducing vascular remodeling through stimulation of H-PGDS-dependent PGD2 release from macrophages, niacin blocks the progression of HySu-induced PAH in rodents (Jia et al., 2020).

The immune microenvironment was significantly altered when PAH rats were exposed to lipopolysaccharide (LPS) and M1 macrophage polarization was increased. By increasing the proportion of M1 macrophages, IL-1 and other inflammatory factors are released, further impairing pulmonary arterial and cardiac function (Guo et al., 2021). The key transcription factor STAT1 can activate signaling cascades leading to macrophage activation and inflammation. METTL3 can upregulate STAT1 expression and promote macrophage M1 polarization by directly methylating STAT1 mRNA (Liu et al., 2019). However, inhibition of METTL3 can inhibit the NF-κB pathway to reduce the macrophage inflammatory response induced by LPS, reducing the progression of PAH (Wang et al., 2019). This shows that inhibition of macrophage inflammatory response can reduce PAH in vascular remodeling. In addition, dysregulation of m^6^A regulators was similarly observed in NK cells, B cells, T cells and Tregs in the stroma (Zheng et al., 2022). However, the mechanism of action between m^6^A and numerous immune cells needs to be further investigated.

In addition, the vascular endothelial growth factor (VEGF) secreted by mast cells in PAH may cause angiogenesis to malfunction, and mast cells around blood vessels produce chymase. It is known that chymase could stimulate vasoconstriction and vascular remodeling by promoting the activation of Ang II, endothelin, and matrix metalloproteases (Qu et al., 2022) (Figure 1). Therefore, inhibiting the secretion of growth factors and cytokines by mast cells may slow the progression of PAH.

In summary, inhibiting the release of inflammatory factors is one of the most important ways to suppress the progression of PAH. In PAH, PASMCs, PAECs, fibroblasts and immune cells are dysfunctional, resulting in pulmonary vascular remodeling. Inflammation could activate the function of immune cells and promote the proliferation of PASMCs and PAECs, leading to pulmonary artery remodeling. Anti-inflammatory therapy may be a viable option for the treatment of severe PAH, which is associated with inflammation and dysregulated immunity.

## 5 Glycolysis and glucose oxidation in PAH

The interaction between metabolism and epigenetics plays a key role in gene expression, cell proliferation, and differentiation. During cellular metabolism, nutrients are absorbed, released, and converted into energy and complex biomolecules. Depending on the availability of nutrients, metabolic products modulate cell signaling and gene expression (Liberti, and Locasale, 2020). A large amount of lactate is produced by anaerobic glycolysis (Zhang et al., 2021), which is originally thought that it was a Warburg effect end product and a metabolic waste product by glycolysis. Nevertheless, lactate is now recognized as an energy source, a signaling molecule, and an immunoregulatory molecule (Bhagat et al., 2019).

**FIGURE 2:**
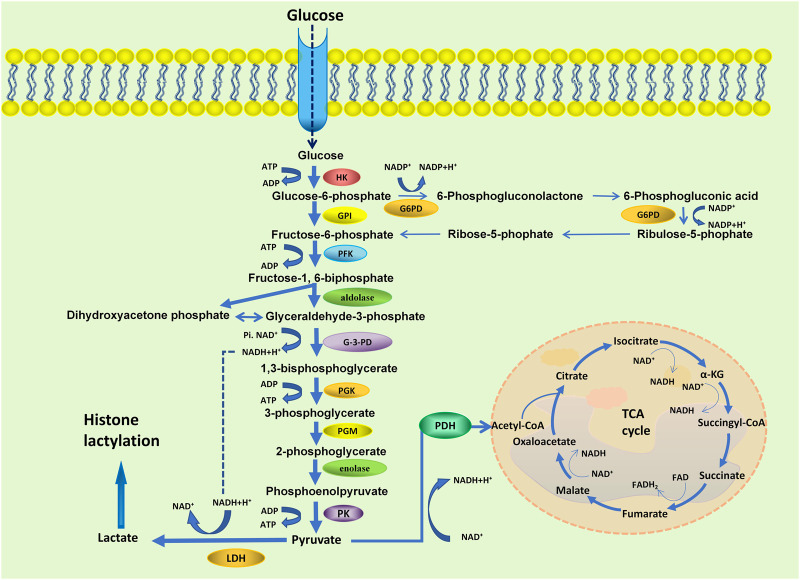
Schematic representation of the glycolysis and the TCA cycle. HK, hexokinase; GPI, phosphoglucose isomerase; G6PD, glycolysis/glucose-6-phosphate dehydrogenase; PFK, 6-phosphfructokinase-1; G-3-PD, glyceraldehyde-3-phosphate dehydrogenase; PGK, phosphoglycerate kinase; PGM, phosphoglycerate mutase; PK, pyruvate kinase; LDH, lactate dehydrogenase; PDH, pyruvate dehydrogenase.

Cellular metabolic reprogramming due to an imbalance between the glycolysis and the citric acid (TCA) cycle, leading to increased histone lactylation (Liberti, and Locasale, 2020). Glucose is first metabolized by glycolysis in tissues to pyruvate, which is then converted to circulating lactate. At the same time, pyruvate can also be oxidized to acetyl-CoA, which participates in the TCA cycle and ATP production (Gustafsson et al., 2007) (Figure 2). During hypoxia, cells reorganize metabolism by suppressing oxidative phosphorylation and increasing glycolysis, which accelerates lactate production (Zhang et al., 2019). Rather than entering the TCA cycle, pyruvate is converted into lactate by cytosolic lactate dehydrogenases (LDHs) in highly glycolytic cells. Finally, as a result of enhanced glycolysis, microenvironments become acidification with increased lactate production.

In PAECs (Cao et al., 2019) and PASMCs (Hernandez-Saavedra et al., 2020) from PAH patients and animal models of PAH, glucose metabolism gradually shifts from mitochondrial oxidative phosphorylation to glycolysis, ultimately leading to elevated lactate levels (Saygin et al., 2017). Meanwhile, evidence suggests that a glycolytic shift increases the proliferation and extracellular matrix (ECM) production of PASMCs, thereby promoting pulmonary vascular remodeling (Kovacs et al., 2019). In addition, glycolysis-related enzymes were increased in PAH lungs, including glycolytic regulator PFKFB2 (6-phosphofructo-2-kinase/fructose-2, 6-biphosphatase) (Zhao et al., 2014) and PFKFB3 (6-phosphofructo-2-kinase/fructose-2,6-bisphosphatase 3). With the increase of glycolysis and lactate level, the expression of PFKFB3 in PASMCs is upregulated, resulting in the proliferation and extracellular collagen synthesis of PASMCs. Studies have shown that PFKFB3 can induce calpain-2 activation and ERK1/2 phosphorylation in pulmonary artery smooth muscle cells, which promote vascular remodeling in PAH. In Sugen/Hypoxia PAH rat model, inhibition of calpain-2 can prevent ERK1/2 activity, and reduces lactate-induced increases of PAH and pulmonary vascular remodeling (Kovacs et al., 2019). Research has also shown that PFKFB3 promotes the production of proinflammatory cytokines and growth factors in PAECs through enhancing endothelial glycolysis. In PAH models, these factors promote inflammation in endothelial cells and the proliferation of PASMCs through autocrine and paracrine pathways (Hernandez-Saavedra et al., 2020).

**FIGURE 3:**
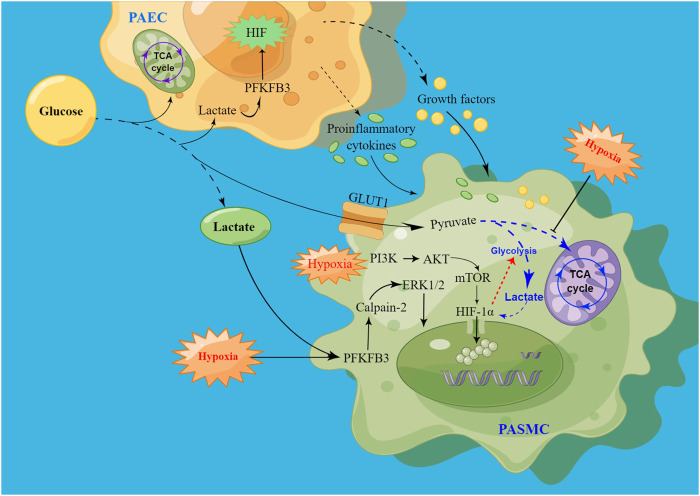
Schematic of signaling pathways driving PASMC proliferation via hypoxia-induced glycolysis. High levels of lactate promote HIF production by increasing PFKFB3 expression, leading to PAECs dysfunction. After injury, PAECs secrete growth factors and proinflammatory cytokines through the paracrine pathway to promote PASMC proliferation. At the same time, PFKFB3 promoted PASMCs proliferation by activating calpain-2 and phosphorylating ERK1/2. In addition, hypoxia promotes HIF release, promotes glycolysis, and inhibits the tricarboxylic acid cycle, thereby increasing lactate levels, and the increase in lactate can also enhance HIF expression. Hypoxia may also promote the onset and development of PAH by activating the PI3K/AKT/mTOR/HIF-1α signaling pathway.

The proliferation of PASMCs is influenced by endothelial dysfunction, hypoxia, inflammation, or mechanical stress, which are augmented by vasoconstrictors, growth factors, and chemokines. Enhanced anaerobic glycolysis can activate HIF, and the overexpression of PFKFB3 also promotes the release of HIF, thus leading to the dysfunction of PAECs (Cao et al., 2019). Hypoxia-induced vasoconstriction is a unique response, and mechanistically, the cellular response to hypoxic conditions is primarily mediated by HIF activation (Yang et al., 2021). Induced vasoconstriction by acute hypoxia results in a reversible increase in pulmonary vascular resistance, whereas prolonged hypoxia promotes PASMCs proliferation and migration, thereby facilitating vascular remodeling and sustained vasoconstriction (Han et al., 2021) (Figure 3).

Under hypoxic conditions, HIF-1 enters the nucleus and associates with hypoxic regulatory genes, thereby enhancing anaerobic glycolysis and further contributing to the hypoxic response (Depping et al., 2008). Several downstream effects activated by HIF-1α are associated with immune escape, and HIF-1α is also an important regulator of macrophage glycolysis metabolism (Mouton et al., 2018). During hypoxia, HIF-1α is increased as a result of oxygen-independent protein synthesis and oxygen-dependent degradation (Kurosawa et al., 2019). During PAH progression, HIF-1α plays an important role in modulating downstream gene transcription (Kurosawa et al., 2019). Studies have shown that HIF-1α expression is upregulated in the pulmonary artery, leading to long-term sustained pulmonary artery constriction and promoting pulmonary artery remodeling (Mouton et al., 2018). Chen et al. have shown that mROS (mitochondrial reactive oxygen species)-dependent HIF-1α accumulation promotes the PASMCs proliferative phenotype (Chen et al., 2022). In addition, high levels of lactate also promote HIF-2α accumulation, leading to PAEC damage (Tang et al., 2018). This suggests that HIF homeostasis is regulated by multiple PTMs that control multiple pathophysiological processes by targeting transcription and translation.

Several signaling pathways may be activated during chronic hypoxia. The mTORC pathway has been shown to be activated in both PASMCs and distal pulmonary arteries from patients with idiopathic PAH (Goncharov et al., 2014). Mechanistically, the mTORC1 pathway activates certain glycolytic enzymes and accelerates glucose metabolism by increasing GLUT1 expression (Liang et al., 2022). With activation of the mTOR-HIF1α axis, the rate of glycolysis is accelerated, resulting in an increase in the production of pyruvate and lactate (Bekkering et al., 2018). In addition, HIF-1α is activated by PI3K/AKT and MAPK/ERK1 pathways in hypoxia conditions (Xu et al., 2016). A classic downstream signaling pathway in PAH, PI3K/AKT activation can promote smooth muscle proliferation in the pulmonary arteries. Previous studies confirmed PAH development by activating the PI3K/AKT/mTOR/HIF-1α signaling pathway (Xiao et al., 2017) (Figure 3). However, the cAMP/PKA signal pathway could suppress mTOR activity (He et al., 2020). Consequently, inhibition of the high expression of HIF and mTOR signaling pathway could suppress pulmonary artery remodeling and the development of PAH.

## 6 Histone lactylation regulates m^6^A affects the development of PAH

Cellular metabolic reactions require glucose and oxygen as substrates. During glycolysis, large amounts of lactate are produced as an energy source to maintain cellular metabolism. Histone lysine lactylation has been shown to be caused by lactate accumulation and regulated by lactate levels. The regulation of gene expression by lactate through histone lactylation modification is a newly discovered epigenetic modification, and a novel PTM has been identified in human and mouse core histones (Bhagat et al., 2019). Histone lactylation is involved in many cellular processes, including translation, metabolism, recombination, and repair (Zhang et al., 2021). Mechanically, lactate is used as a substrate to generate lactyl-CoA for lysine lactylation on histones, a process that regulates gene expression in a variety of pathophysiological conditions (Zhang et al., 2019). Meanwhile, in terms of transcription and antigenic variation, chromatin repression or induction is determined by the PTM status of core histones (Stillman, 2018).

In addition to their critical function in signal transduction and cellular metabolism, PTMs also play a key role in regulating protein conformation, stability and function (Zhang et al., 2021). Several factors were associated with PASMCs and PAECs proliferation, including lactate metabolism, oxidative stress response, HIF-1 pathway and PTMs. A number of studies have shown that glycolysis plays a critical role in PASMC proliferation, and inhibition of glycolysis can inhibit PASMC proliferation and migration and also reverse PAH in animal models (Xiao et al., 2017). Chen et al. found that mROS-mediated HIF-1α-driven glycolysis promotes pulmonary artery remodeling. Mechanistically, lactate accumulation increases histone lactylation at HIF-1α targets linked to proliferative phenotype (Chen et al., 2022).

Lactate in the intracellular environment can promote the lactylation of histone H3 on the promoters of homeostatic genes, which activates their expression (Zhang et al., 2019). A study found that METTL3 expression was upregulated in tumor-infiltrating myeloid cells (TIMs) and associated with poor prognosis. Meanwhile, study confirmed that lactylation was indeed present in METTL3, and H3K18la was enriched in the promoter regions of METTL3. In a mechanical manner, lactate promotes METTL3 transcription by modifying H3K18la. Lactate accumulated in the tumor microenvironment potently promoted METTL3 upregulation in TIMs through H3K18la, and lactylation of METTL3 in TIMs promoted m^6^A-mediated immunosuppression (Xiong et al., 2022). In addition, the “CCCH” zinc finger domains (ZFDs) of the METTL3 protein can be directly lactylated, which via the METTL3-JAK1-STAT3 signaling pathway. METTL3 was bound and enhances m^6^A modification of target RNA and promotes the expression of downstream immunosuppressive effector molecules like iNOS, IL-6, and IL-10 (Kumagai et al., 2022). This suggests that lactate could promote METTL3 expression through H3K18la modification, thereby affecting downstream signaling and gene expression.

METTL3 expression is upregulated in hypoxia-induced PASMCs, which promotes pulmonary artery remodeling through the METTL3/YTHDF2/PTEN axis (Qin et al., 2021). Meanwhile, studies have shown that lactate promotes PASMC proliferation through histone lactylation modification. H3K18laChIP-seq analysis of PDH kinase 1 (PDK1) and PDK2 silenced hypoxic PASMCs revealed that the density of H3K18la around the HIF-1α peak was also reduced (Chen et al., 2022). This suggests that both histone lactylation and METTL3 play important roles in PAH. However, the specific role of H3K18la and METTL3 in PAH is still unclear and needs to be further explored, which will also provide an important basis for the treatment of PAH.

**FIGURE 4:**
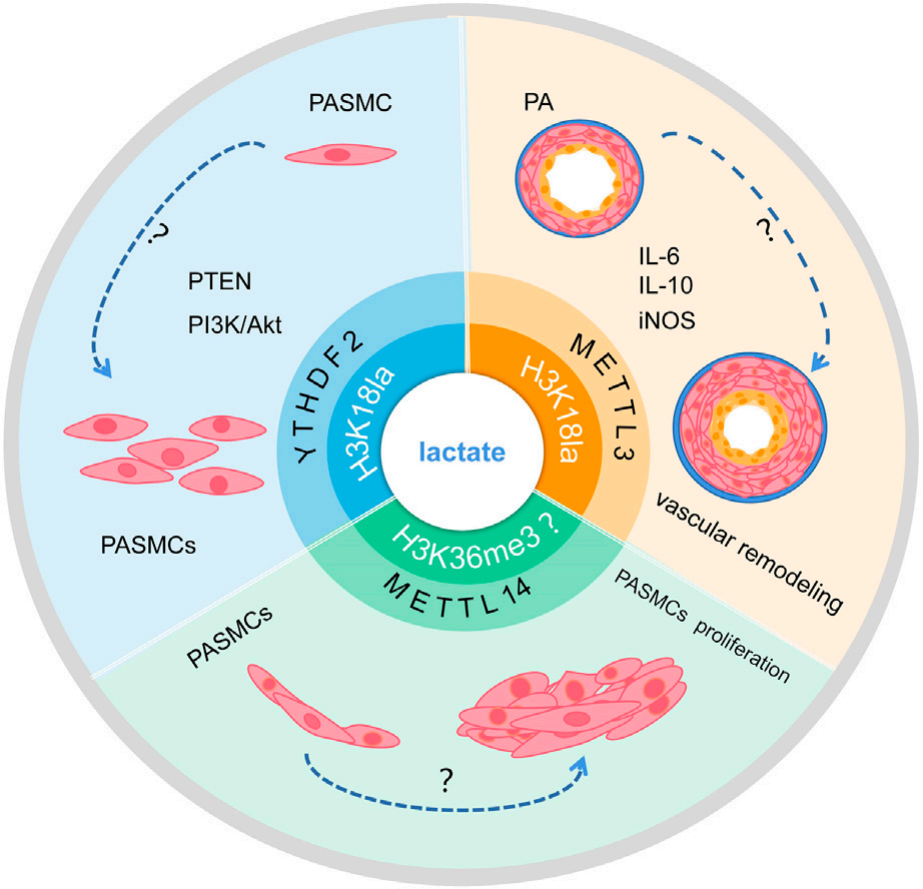
Schematic of the hypothesis that lactate regulates m^6^A to affect PAH development via histone lactylation modification. Lactate may promote transcription of RNA METTL3 and YTHDF2 through H3K18la modification, and whether it may further influence PAH progression remains to be studied. In addition, whether lactate can affect the transcription of METTL14 and interfere with PASMC proliferation through H3K36me3 modification remains to be investigated.

The metabolic dynamics of glucose and lactate levels change to regulate histone lactylation (Varner et al., 2020). Previous studies have shown that histone lactylation may contribute to tumor growth by increasing YTHDF2 transcription. One study confirmed that H3K18la enrichment is present at the promoter of YTHDF2, transcription of YTHDF2 is regulated by H3K18la, and glycolysis inhibitors reduced this enrichment (Yu et al., 2021). Meanwhile, another study showed that the translation and expression of LDHB are decreased by YTHDF2, which inhibits aerobic glycolysis and cell proliferation by promoting mRNA degradation (Huang et al., 2020; Qing et al., 2021). YTHDF2 is upregulated and expressed in PAH and inhibited YTHDF2 can prevent hypoxia-induced PASMC proliferation. However, the specific role of histone lactylation and YTHDF2 in PAH needs to be further explored (Figure 4).

In addition, modifications of m^6^A are enriched around H3K36me3 peaks, and are reduced globally when H3K36me3 is depleted in the cell, this indicated that loss of H3K36me3 reduces m^6^A methylation. H3K36me3 and m^6^A modifications overlapped well with METTL14 binding sites on RNA, according to distance analysis. In terms of mechanism, METTL14 recognizes and binds H3K36me3 directly, m^6^A co-transcriptionally deposited by delivering the m^6^A methyltransferase complex (MTC) on actively transcribed nascent RNAs (Huang et al., 2019). Evidence shown that METTL14 is upregulated expressed in PAH and inhibited METTL14 can prevent hypoxia-induced PASMCs proliferation (Zhou et al., 2021). However, The mechanism of action between lactate and H3K36me3 remains unclear. The target mechanism of histone lactylation involved in the methylation modification of METTL14 to regulate the occurrence and development of PAH requires further study.

## 7 The immune microenvironment disrupted by histone lactylation and promotes the development of PAH

Histone lysine lactylation is involved in the regulation of gene expression by affecting mRNA splicing, translation, processing, and degradation. A growing body of evidence suggests that lactate regulates both innate and adaptive immune cells and affects significant changes in gene expression in a unique way (Zhang et al., 2019). According to lactate homeostasis, lactate is vital in fine-tuning cellular metabolism by regulating extracellular metabolism, and the function of lactate metabolism is further emphasized by energy homeostasis (Lagarde et al., 2021). In addition to playing a role in metabolism, lactate or signal molecules are involved in a variety of physiological and pathological processes. Lactate shuttles between and within cells to accomplish its effects and affects cell function. This shows that connect histone lactylation metabolism and the importance of epigenetic process.

Lactate is an active signal that regulates immune cells, metabolically reprogramming them to regulate their function (Lee et al., 2018). Histone lactylation has been shown to modulate immune responses and play important biological roles in the immune system. Lactate promotes the release of pro-inflammatory cytokines by regulating a variety of immune cell functions. Lactate can accumulate in response to inflammation or hypoperfusion. Studies have shown that lactate is a powerful amplifier of inflammation in arthritis (Souto-Carneiro et al., 2020). In PAH, an altered immune system contributes significantly to pulmonary vascular remodelling by promoting inflammatory cell recruitment and autoimmune dysfunction (Xu et al., 2021).

Most immunometabolic studies have focused on tumour-associated macrophages in cancer or abnormal B and T lymphocyte function in autoimmune diseases. Several studies have shown that lactate suppresses the proliferation, migration and function of T cells (Brand et al., 2016). Extracellular lactate levels are sensed by T cells, causing intracellular signalling and altering cell function and homeostasis. Excessive lactate inhibits T-cell mediated immune responses (Watson et al., 2021). By aerobic oxidative metabolism, glucose is mainly metabolised to carbon dioxide by resting T cells, whereas activated cytotoxic T cells utilise glycolysis and produce lactate for energy and biosynthesis (Fischer et al., 2007).

**FIGURE 5:**
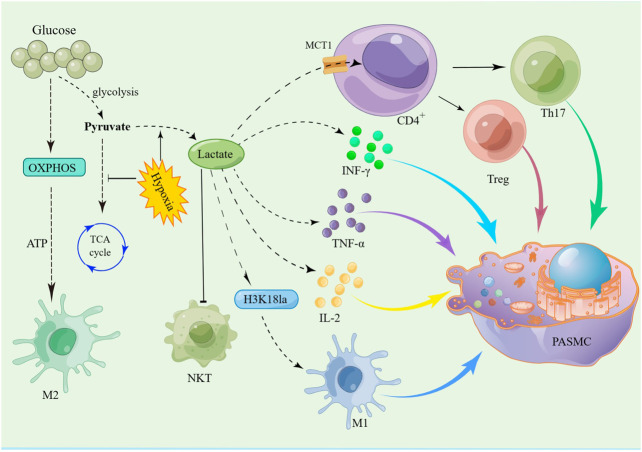
Schematic of the hypothesis that lactate promotes PASMC proliferation by disrupting the immune microenvironment via histone lactylation modification. Glycolysis and the TCA cycle are the major metabolic processes of glucose in the body. When oxygen is adequate, cells produce energy primarily through the TCA cycle. However, in hypoxia, glucose is metabolized by glycolysis to produce large amounts of lactate. Lactate is transported via MCT1 to CD4^+^ T cells, which then promote the differentiation of Th17 cells and the expansion of Treg cells. At the same time, the increase in lactate promotes the secretion of IFN-γ, IL-2 and TNF-α by immune cells, which promotes PASMC proliferation by activating downstream signalling pathways. In addition, lactate promotes the release of IL-1 through histone lactylation modification of M1 macrophages, thereby promoting the proliferation of PASMC.

Lactate signalling in CD4^+^ T cells promotes Th17 cell differentiation and suppresses T cell migration and trafficking (Pucino et al., 2019). Lactate enters CD4^+^ T cells via MCT1, through LDHB into pyruvate, promote TCA cycle, decrease T-cell glycolysis, inhibits CD4^+^ T cell proliferation, induces effector T cell dysfunction (Kaushik et al., 2019), favors Treg expansion, and maintains their suppressive function (Watson et al., 2021). A link has been established between aerobic glycolysis and cytokine production. Several studies have shown that glycolytic enzymes are involved in the production of cytokines. *Ex vivo* T-cell activation assays have shown that lactate stimulates the secretion of cytokines such as IFN-γ, IL-2 and TNF-α (Wen et al., 2021). In addition, other studies found that the high lactate microenvironment decreased IFN-g production and inhibited NKT cell proliferation, survival and effector function (Kumar et al., 2019) (Figure 5).

An important mechanism for the induction of macrophage plasticity is the modulation of phenotypic stability and epigenetic dynamics in the context of inflammation, autoimmune responses and cancer. Under physiological or pathological conditions, epigenetic modification may form an integrated pathway during lactate-induced cell polarisation (Bekkering et al., 2018). Previous studies have shown that glycolysis and oxidative phosphorylation (OXPHOS) are closely linked to macrophage polarisation. There are two types of activated macrophages: pro-inflammatory M1 macrophages rely primarily on glycolysis, whereas reparative and immunoregulatory M2 macrophages rely on OXPHOS (Watanabe et al., 2018) (Figure 5). Thus, these factors that affect macrophage metabolism may disrupt M1/M2 homeostasis and exacerbate inflammation.

PAH is the result of a variety of factors and one of the most important is the imbalance of the immune microenvironment. Lactate can increase the expression of pro-inflammatory cytokines and regulate macrophage polarisation both *in vivo* and *in vitro*. Boutens et al. found that in human cell lines, hypoxia and glucose supplementation increased intracellular lactate levels and upregulated the expression of histone lactylation, and in particular promoted histone H3K18 lactylation (Sun et al., 2021), thereby promoting the polarisation of M1-type macrophages (Boutens et al., 2018) (Figure 5). The research showed that lactate production is required for proper histone lactylation, which induces gene expression and maintains homeostasis by promoting an M2-like phenotype in the late stages of M1 macrophage polarisation. In the M1 macrophage polarisation model, ChIP-seq showed that H3K18la was enriched at specific genes. When M1 macrophages are polarised by infection, this is characterised by increased histone lactylation in promoter regions and leads to the expression of homeostatic genes (Zhang et al., 2019). One line of clinical evidence suggests that the expression of H3K18 in peripheral blood monocytes is strongly correlated with the severity of critically ill patients. Therefore, H3K18 is a very promising biomarker (Chu et al., 2021).

Endothelial dysfunction accompanied by glycolysis increase metabolic changes in the pathophysiology, PAH is of great importance. Recent studies have shown that lactate increases the acetylation and lactylation of high mobility group protein B1 (HMGB1), and enhances its release from macrophages through exosomes. In addition, lactate inhibits the steady state and promotes vascular permeability, which induces vascular endothelial cell injury (Yang et al., 2022). Meanwhile, from *in vitro* cultured PASMC, HMGB1 by increasing the endoplasmic reticulum stress-related protein PERK and ATF4 reduce HIPK2 expression, increase SIAH2 expression, thus promoting PASMC proliferation and migration. Through glycyrrhizic acid interference, HMGB1 can reduce the development of PAH (Zhang et al., 2023). Glucose enters the cytoplasm through the glucose transporter 1 (GLUT1) and is metabolized through the pathways of glycolysis and the tricarboxylic acid cycle. Overexpression of the primary macrophage GLUT1 enhances glycolysis and pro-inflammatory cytokine release. Similarly, lacking GLUT1 of macrophages promoted M2 polarization (Freemerman et al., 2019). It has been shown that GLUT-1 is over-expressed in PAs and PASMCs in an animal model of MCT-induced PAH (Li et al., 2019). In addition, studies have shown that the increase of pyruvate kinase M2 (PKM2) protein expression in PAH can promote the phosphorylation of ERK1/2 and further upregulate the expression of key glycolytic enzymes LDHA and GLUT1, thereby participating in vascular remodeling in PAH. However, increasing shikonin decreased the protein level of PKM2, decreased the phosphorylation level of ERK1/2 and the expression level of GLUT1 protein, and inhibited the progression of PAH (Li et al., 2023).

A hypoxia-induced adaptive response is initiated by HIF-1, which increases or represses the expression of genes regulating vascular tone, autophagic response, cell metabolism, and proliferation. HIF-1 could enhance the transcription of a glycolysis and pro-inflammatory M1 gene profile (Boutens et al., 2018). Lactate, as a promoter of angiogenesis, increases angiogenesis through HIF-1α stabilization to promote the expression of VEGF (Depping et al., 2008) Furthermore, studies have shown that the progression of PAH is due to VEGF (Wang et al., 2022) and Arginase (Arg) (Ji et al., 2022) overexpression. Hypoxia induces changes in the subcellular distribution of nuclear proteins and significantly promotes the activation of EGFR signaling. The phosphorylation modification of EGFR increases the sensitivity of vascular cells to Ca^2+^, leading to enhanced vasoconstriction and the development of pulmonary vascular remodeling, whereas injection of EGFR inhibitors can improve pulmonary artery remodeling in MCT-induced PH rats (Wang et al., 2022). EGFR can activate downstream ERK, and ERK phosphorylation can activate HIF-1. In addition, lactate induced M2 macrophage polarization can be attributed to the activator ERK of the STAT3 signaling pathway as well as increased VEGF and Arg-1 expression (Mu et al., 2018).

In conclusion, lactate accumulation and histone lactylation contribute to the development of immunotherapy (Cascone et al., 2018). Several studies have shown that there is some correlation between immune cells and glucose metabolites. Therapies targeting immune metabolism are in the early stages of development. However, the mechanism of their interaction, whether through direct or indirect signaling pathways, remains unclear and needs to be further explored. In this review, we bridge the gap between histone lactylation and the immune microenvironment for the first time, providing new insights into PAH research.

## 8 Conclusion

PAH is a serious cardiovascular disease that results from a complex mechanism involving many cellular and molecular interactions, and recent studies have shown that lactate plays an important role in PAH. While impressive progress has been made, there are still many questions that remain unanswered. Specifically, lactate can affect m^6^A through histone lactylation modification, thereby altering transcription and translation of mRNA, which in turn affects cell growth and metabolism. In addition, lactate may also affect the immune microenvironment by regulating the number and function of immune cells, thereby affecting the disease course of patients with PAH.

Existing literature shows that in almost all proteins involved in at least one regulatory PTM. Lactylated proteins are widely involved in PTMs and protein turnover, and are involved in chaperones, ribosomal structure, and biogenesis (Zhang et al., 2021). Lactate regulates cellular metabolism through histone lactylation-mediated gene expression. In addition, lactate has been shown to play an important role in angiogenesis, energy supply, immunosuppression, and epigenetic regulation (Jiang et al., 2021). The lysine lactylation in core histones is a novel type of histone mark. So far, 28 lactylation sites have been identified, H3, H4, H2A, and H2B are among the sites for lactylation on core histones (Zhang et al., 2019). The discovery of novel signaling pathways, transcription factors, biomarkers and metabolic mediators of PAH, as well as intersections that may aid in the development of effective targeted therapies, is essential.

Investigating the biological mechanisms behind the onset and progression of PAH is critical to more effectively treating the disease, improving its prognosis and developing effective strategies to reverse it. With the discovery of lactylation, the historical role of lactate has been re-examined from a biological and functional perspective. Therapeutic strategies targeting lactate metabolism are becoming increasingly useful and promising. Because lactate stimulates histone lactylation modifications and contributes to gene expression, advancing our knowledge of the physiopathology of PAH with histone lactylation modification is likely to fill an important knowledge gap.

## References

[B1] Al-Omary M. S. Sugito S. Boyle A. J. Sverdlov A. L. Collins N. J. (2020). Pulmonary hypertension due to left heart disease: diagnosis, pathophysiology, and therapy. Hypertension 75 (6), 1397–1408. 10.1161/HYPERTENSIONAHA.119.14330 32336230

[B2] Badesch D. B. Raskob G. E. Elliott C. G. Krichman A. M. Farber H. W. Frost A. E. (2010). Pulmonary arterial hypertension: baseline characteristics from the REVEAL registry. Chest 137 (2), 376–387. 10.1378/chest.09-1140 19837821

[B3] Bekkering S. Arts R. J. W. Novakovic B. Kourtzelis I. van der Heijden C. Li Y. (2018). Metabolic induction of trained immunity through the mevalonate pathway. Cell 172 (1-2), 135–146. 10.1016/j.cell.2017.11.025 29328908

[B4] Bhagat T. D. Von Ahrens D. Dawlaty M. Zou Y. Baddour J. Achreja A. (2019). Lactate-mediated epigenetic reprogramming regulates formation of human pancreatic cancer-associated fibroblasts. Elife 8, e50663. 10.7554/eLife.50663 31663852 PMC6874475

[B5] Boucly A. Savale L. Jais X. Bauer F. Bergot E. Bertoletti L. (2021). Association between initial treatment strategy and long-term survival in pulmonary arterial hypertension. Am. J. Respir. Crit. Care Med. 204 (7), 842–854. 10.1164/rccm.202009-3698OC 34185620

[B6] Boutens L. Hooiveld G. J. Dhingra S. Cramer R. A. Netea M. G. Stienstra R. (2018). Unique metabolic activation of adipose tissue macrophages in obesity promotes inflammatory responses. Diabetologia 61 (4), 942–953. 10.1007/s00125-017-4526-6 29333574 PMC6448980

[B7] Brand A. Singer K. Koehl G. E. Kolitzus M. Schoenhammer G. Thiel A. (2016). LDHA-associated lactic acid production blunts tumor immunosurveillance by T and NK cells. Cell Metab. 24 (5), 657–671. 10.1016/j.cmet.2016.08.011 27641098

[B8] Cao Y. Zhang X. Wang L. Yang Q. Ma Q. Xu J. (2019). PFKFB3-mediated endothelial glycolysis promotes pulmonary hypertension. Proc. Natl. Acad. Sci. U. S. A. 116 (27), 13394–13403. 10.1073/pnas.1821401116 31213542 PMC6613097

[B9] Cascone T. McKenzie J. A. Mbofung R. M. Punt S. Wang Z. Xu C. (2018). Increased tumor glycolysis characterizes immune resistance to adoptive T cell therapy. Cell Metab. 27 (5), 977–987. 10.1016/j.cmet.2018.02.024 29628419 PMC5932208

[B10] Chu Y. Xiangli X. Xiao W. (2015). Regulatory T cells protect against hypoxia-induced pulmonary arterial hypertension in mice. Mol. Med. Rep. 11 (4), 3181–3187. 10.3892/mmr.2014.3106 25523119

[B11] Chen G. Zuo S. Tang J. Zuo C. Jia D. Liu Q. (2018). Inhibition of CRTH2-mediated Th2 activation attenuates pulmonary hypertension in mice. J. Exp. Med. 215 (8), 2175–2195. 10.1084/jem.20171767 29970474 PMC6080901

[B12] Chen H. Gu L. Orellana E. A. Wang Y. Guo J. Liu Q. (2020). METTL4 is an snRNA m(6)Am methyltransferase that regulates RNA splicing. Cell Res. 30 (6), 544–547. 10.1038/s41422-019-0270-4 31913360 PMC7264358

[B13] Chen J. Zhang M. Liu Y. Zhao S. Wang Y. Wang M. (2022). Histone lactylation driven by mROS-mediated glycolytic shift promotes hypoxic pulmonary hypertension. J. Mol. Cell Biol. 14 (12), mjac073. 10.1093/jmcb/mjac073 PMC10175659 36564027

[B14] Chu X. Di C. Chang P. Li L. Feng Z. Xiao S. (2021). Lactylated histone H3K18 as a potential biomarker for the diagnosis and predicting the severity of septic shock. Front. Immunol. 12, 786666. 10.3389/fimmu.2021.786666 35069560 PMC8773995

[B15] Depping R. Steinhoff A. Schindler S. G. Friedrich B. Fagerlund R. Metzen E. (2008). Nuclear translocation of hypoxia-inducible factors (HIFs): involvement of the classical importin alpha/beta pathway. Biochim. Biophys. Acta 1783 (3), 394–404. 10.1016/j.bbamcr.2007.12.006 18187047

[B16] Diebold I. Hennigs J. K. Miyagawa K. Li C. G. Nickel N. P. Kaschwich M. (2015). BMPR2 preserves mitochondrial function and DNA during reoxygenation to promote endothelial cell survival and reverse pulmonary hypertension. Cell Metab. 21 (4), 596–608. 10.1016/j.cmet.2015.03.010 25863249 PMC4394191

[B17] Du H. Zhao Y. He J. Zhang Y. Xi H. Liu M. (2016). YTHDF2 destabilizes m(6)A-containing RNA through direct recruitment of the CCR4-NOT deadenylase complex. Nat. Commun. 7, 12626. 10.1038/ncomms12626 27558897 PMC5007331

[B18] Efremova M. Vento-Tormo M. Teichmann S. A. Vento-Tormo R. (2020). CellPhoneDB: inferring cell-cell communication from combined expression of multi-subunit ligand-receptor complexes. Nat. Protoc. 15 (4), 1484–1506. 10.1038/s41596-020-0292-x 32103204

[B19] Elkord E. (2009). Frequency of human T regulatory cells in peripheral blood is significantly reduced by cryopreservation. J. Immunol. Methods 347 (1-2), 87–90. 10.1016/j.jim.2009.06.001 19538964

[B20] Fei Q. Zou Z. Roundtree I. A. Sun H. L. He C. (2020). YTHDF2 promotes mitotic entry and is regulated by cell cycle mediators. PLoS Biol. 18 (4), e3000664. 10.1371/journal.pbio.3000664 32267835 PMC7170294

[B21] Fischer K. Hoffmann P. Voelkl S. Meidenbauer N. Ammer J. Edinger M. (2007). Inhibitory effect of tumor cell-derived lactic acid on human T cells. Blood 109 (9), 3812–3819. 10.1182/blood-2006-07-035972 17255361

[B22] Florentin J. Coppin E. Vasamsetti S. B. Zhao J. Tai Y. Y. Tang Y. (2018). Inflammatory macrophage expansion in pulmonary hypertension depends upon mobilization of blood-borne monocytes. J. Immunol. 200 (10), 3612–3625. 10.4049/jimmunol.1701287 29632145 PMC5940510

[B23] Freemerman A. J. Zhao L. Pingili A. K. Teng B. Cozzo A. J. Fuller A. M. (2019). Myeloid slc2a1-deficient murine model revealed macrophage activation and metabolic phenotype are fueled by GLUT1. J. Immunol. 202 (4), 1265–1286. 10.4049/jimmunol.1800002 30659108 PMC6360258

[B24] Galie N. Humbert M. Vachiery J. L. Gibbs S. Lang I. Torbicki A. (2016). 2015 ESC/ERS guidelines for the diagnosis and treatment of pulmonary hypertension. Rev. Esp. Cardiol. Engl. Ed. 69 (2), 177. 10.1016/j.rec.2016.01.002 26837729

[B25] Gao Y. Pei G. Li D. Li R. Shao Y. Zhang Q. C. (2019). Multivalent m(6)A motifs promote phase separation of YTHDF proteins. Cell Res. 29 (9), 767–769. 10.1038/s41422-019-0210-3 31388144 PMC6796879

[B26] Goncharov D. A. Kudryashova T. V. Ziai H. Ihida-Stansbury K. DeLisser H. Krymskaya V. P. (2014). Mammalian target of rapamycin complex 2 (mTORC2) coordinates pulmonary artery smooth muscle cell metabolism, proliferation, and survival in pulmonary arterial hypertension. Circulation 129 (8), 864–874. 10.1161/CIRCULATIONAHA.113.004581 24270265 PMC3968690

[B27] Guihaire J. Deuse T. Wang D. Spin J. M. Blankenberg F. G. Fadel E. (2021). Immunomodulation therapy using tolerogenic macrophages in a rodent model of pulmonary hypertension. Stem Cells Dev. 30 (10), 515–525. 10.1089/scd.2021.0007 33726521

[B28] Guo L. Qin G. Cao Y. Yang Y. Dai S. Wang L. (2021). Regulation of the immune microenvironment by an NLRP3 inhibitor contributes to attenuation of acute right ventricular failure in rats with pulmonary arterial hypertension. J. Inflamm. Res. 14, 5699–5711. 10.2147/JIR.S336964 34754216 PMC8572093

[B29] Guo X. Li K. Jiang W. Hu Y. Xiao W. Huang Y. (2020). RNA demethylase ALKBH5 prevents pancreatic cancer progression by posttranscriptional activation of PER1 in an m6A-YTHDF2-dependent manner. Mol. Cancer 19 (1), 91. 10.1186/s12943-020-01158-w 32429928 PMC7236181

[B30] Gustafsson J. Eriksson J. Marcus C. (2007). Glucose metabolism in human adipose tissue studied by 13C-glucose and microdialysis. Scand. J. Clin. Lab. Invest. 67 (2), 155–164. 10.1080/00365510600995259 17365995

[B31] Han X. J. Zhang W. F. Wang Q. Li M. Zhang C. B. Yang Z. J. (2021). HIF-1α promotes the proliferation and migration of pulmonary arterial smooth muscle cells via activation of Cx43. J. Cell Mol. Med. 25 (22), 10663–10673. 10.1111/jcmm.17003 34698450 PMC8581339

[B32] Harbaum L. Renk E. Yousef S. Glatzel A. Luneburg N. Hennigs J. K. (2016). Acute effects of exercise on the inflammatory state in patients with idiopathic pulmonary arterial hypertension. BMC Pulm. Med. 16 (1), 145. 10.1186/s12890-016-0301-6 27835955 PMC5106767

[B33] Harbaum L. Rhodes C. J. Wharton J. Lawrie A. Karnes J. H. Desai A. A. (2022). Mining the plasma proteome for insights into the molecular pathology of pulmonary arterial hypertension. Am. J. Respir. Crit. Care Med. 205 (12), 1449–1460. 10.1164/rccm.202109-2106OC 35394406 PMC9875902

[B34] Hautefort A. Mendes-Ferreira P. Sabourin J. Manaud G. Bertero T. Rucker-Martin C. (2019). Bmpr2 mutant rats develop pulmonary and cardiac characteristics of pulmonary arterial hypertension. Circulation 139 (7), 932–948. 10.1161/CIRCULATIONAHA.118.033744 30586714

[B35] He Y. Zuo C. Jia D. Bai P. Kong D. Chen D. (2020). Loss of DP1 aggravates vascular remodeling in pulmonary arterial hypertension via mTORC1 signaling. Am. J. Respir. Crit. Care Med. 201 (10), 1263–1276. 10.1164/rccm.201911-2137OC 31917615 PMC7233340

[B36] Hernandez-Saavedra D. Sanders L. Freeman S. Reisz J. A. Lee M. H. Mickael C. (2020). Stable isotope metabolomics of pulmonary artery smooth muscle and endothelial cells in pulmonary hypertension and with TGF-beta treatment. Sci. Rep. 10 (1), 413. 10.1038/s41598-019-57200-5 31942023 PMC6962446

[B37] Hong K. H. Lee Y. J. Lee E. Park S. O. Han C. Beppu H. (2008). Genetic ablation of the BMPR2 gene in pulmonary endothelium is sufficient to predispose to pulmonary arterial hypertension. Circulation 118 (7), 722–730. 10.1161/CIRCULATIONAHA.107.736801 18663089 PMC3920834

[B38] Hu L. Wang J. Huang H. Yu Y. Ding J. Yu Y. (2021). YTHDF1 regulates pulmonary hypertension through translational control of MAGED1. Am. J. Respir. Crit. Care Med. 203 (9), 1158–1172. 10.1164/rccm.202009-3419OC 33465322

[B39] Huang H. Weng H. Zhou K. Wu T. Zhao B. S. Sun M. (2019). Histone H3 trimethylation at lysine 36 guides m(6)A RNA modification co-transcriptionally. Nature 567 (7748), 414–419. 10.1038/s41586-019-1016-7 30867593 PMC6438714

[B40] Huang T. Liu Z. Zheng Y. Feng T. Gao Q. Zeng W. (2020). YTHDF2 promotes spermagonial adhesion through modulating MMPs decay via m(6)A/mRNA pathway. Cell Death Dis. 11 (1), 37. 10.1038/s41419-020-2235-4 31959747 PMC6971064

[B41] Huertas A. Phan C. Bordenave J. Tu L. Thuillet R. Le Hiress M. (2016). Regulatory T cell dysfunction in idiopathic, heritable and connective tissue-associated pulmonary arterial hypertension. Chest 149 (6), 1482–1493. 10.1016/j.chest.2016.01.004 26836928

[B42] Humbert M. Kovacs G. Hoeper M. M. Badagliacca R. Berger R. M. F. Brida M. (2022). 2022 ESC/ERS Guidelines for the diagnosis and treatment of pulmonary hypertension. Eur. Heart J. 43 (38), 3618–3731. 10.1093/eurheartj/ehac237 36017548

[B43] Isobe S. Kataoka M. Endo J. Moriyama H. Okazaki S. Tsuchihashi K. (2019). Endothelial-Mesenchymal transition drives expression of CD44 variant and xCT in pulmonary hypertension. Am. J. Respir. Cell Mol. Biol. 61 (3), 367–379. 10.1165/rcmb.2018-0231OC 30897333

[B44] Jain D. Puno M. R. Meydan C. Lailler N. Mason C. E. Lima C. D. (2018). Ketu mutant mice uncover an essential meiotic function for the ancient RNA helicase YTHDC2. Elife 7, e30919. 10.7554/eLife.30919 29360036 PMC5832417

[B45] Ji L. Su S. Xin M. Zhang Z. Nan X. Li Z. (2022). Luteolin ameliorates hypoxia-induced pulmonary hypertension via regulating HIF-2α-Arg-NO axis and PI3K-AKT-eNOS-NO signaling pathway. Phytomedicine 104, 154329. 10.1016/j.phymed.2022.154329 35843187

[B46] Jia D. Bai P. Wan N. Liu J. Zhu Q. He Y. (2020). Niacin attenuates pulmonary hypertension through H-pgds in macrophages. Circ. Res. 127 (10), 1323–1336. 10.1161/CIRCRESAHA.120.316784 32912104

[B47] Jiang J. Huang D. Jiang Y. Hou J. Tian M. Li J. (2021). Lactate modulates cellular metabolism through histone lactylation-mediated gene expression in non-small cell lung cancer. Front. Oncol. 11, 647559. 10.3389/fonc.2021.647559 34150616 PMC8208031

[B48] Kaushik D. K. Bhattacharya A. Mirzaei R. Rawji K. S. Ahn Y. Rho J. M. (2019). Enhanced glycolytic metabolism supports transmigration of brain-infiltrating macrophages in multiple sclerosis. J. Clin. Invest. 129 (8), 3277–3292. 10.1172/JCI124012 31112527 PMC6668690

[B49] Ke S. Alemu E. A. Mertens C. Gantman E. C. Fak J. J. Mele A. (2015). A majority of m6A residues are in the last exons, allowing the potential for 3' UTR regulation. Genes Dev. 29 (19), 2037–2053. 10.1101/gad.269415.115 26404942 PMC4604345

[B50] Kovacs L. Cao Y. Han W. Meadows L. Kovacs-Kasa A. Kondrikov D. (2019). PFKFB3 in smooth muscle promotes vascular remodeling in pulmonary arterial hypertension. Am. J. Respir. Crit. Care Med. 200 (5), 617–627. 10.1164/rccm.201812-2290OC 30817168 PMC6727156

[B51] Kumagai S. Koyama S. Itahashi K. Tanegashima T. Lin Y. T. Togashi Y. (2022). Lactic acid promotes PD-1 expression in regulatory T cells in highly glycolytic tumor microenvironments. Cancer Cell 40 (2), 201–218.e9. 10.1016/j.ccell.2022.01.001 35090594

[B52] Kumar A. Pyaram K. Yarosz E. L. Hong H. Lyssiotis C. A. Giri S. (2019). Enhanced oxidative phosphorylation in NKT cells is essential for their survival and function. Proc. Natl. Acad. Sci. U. S. A. 116 (15), 7439–7448. 10.1073/pnas.1901376116 30910955 PMC6462103

[B53] Kurosawa R. Satoh K. Kikuchi N. Kikuchi H. Saigusa D. Al-Mamun M. E. (2019). Identification of celastramycin as a novel therapeutic agent for pulmonary arterial hypertension. Circ. Res. 125 (3), 309–327. 10.1161/CIRCRESAHA.119.315229 31195886

[B54] Lagarde D. Jeanson Y. Barreau C. Moro C. Peyriga L. Cahoreau E. (2021). Lactate fluxes mediated by the monocarboxylate transporter-1 are key determinants of the metabolic activity of beige adipocytes. J. Biol. Chem. 296, 100137. 10.1074/jbc.RA120.016303 33268383 PMC7949083

[B55] Lee Y. S. Kim T. Y. Kim Y. Lee S. H. Kim S. Kang S. W. (2018). Microbiota-Derived lactate accelerates intestinal stem-cell-mediated epithelial development. Cell Host Microbe 24 (6), 833–846. 10.1016/j.chom.2018.11.002 30543778

[B56] Li B. He W. Ye L. Zhu Y. Tian Y. Chen L. (2019). Targeted delivery of sildenafil for inhibiting pulmonary vascular remodeling. Hypertension 73 (3), 703–711. 10.1161/HYPERTENSIONAHA.118.11932 30636546

[B57] Li J. Xie H. Ying Y. Chen H. Yan H. He L. (2020a). YTHDF2 mediates the mRNA degradation of the tumor suppressors to induce AKT phosphorylation in N6-methyladenosine-dependent way in prostate cancer. Mol. Cancer 19 (1), 152. 10.1186/s12943-020-01267-6 33121495 PMC7599101

[B58] Li W. Chen W. Peng H. Xiao Z. Liu J. Zeng Y. (2023). Shikonin improves pulmonary vascular remodeling in monocrotaline-induced pulmonary arterial hypertension via regulation of PKM2. Mol. Med. Rep. 27 (3), 60. Epub 2023 Feb 3. 10.3892/mmr.2023.12947 36734266 PMC9936259

[B59] Li Z. Li F. Peng Y. Fang J. Zhou J. (2020b). Identification of three m6A-related mRNAs signature and risk score for the prognostication of hepatocellular carcinoma. Cancer Med. 9 (5), 1877–1889. 10.1002/cam4.2833 31943856 PMC7050095

[B60] Li Z. Qian P. Shao W. Shi H. He X. C. Gogol M. (2018). Suppression of m(6)A reader Ythdf2 promotes hematopoietic stem cell expansion. Cell Res. 28 (9), 904–917. 10.1038/s41422-018-0072-0 30065315 PMC6123498

[B61] Liang Y. Wang X. Wang H. Yang W. Yi P. Soong L. (2022). IL-33 activates mTORC1 and modulates glycolytic metabolism in CD8(+) T cells. Immunology 165 (1), 61–73. 10.1111/imm.13404 34411293 PMC9112898

[B62] Liberti M. V. Locasale J. W. (2020). Histone lactylation: A new role for glucose metabolism. Trends Biochem. Sci. 45 (3), 179–182. 10.1016/j.tibs.2019.12.004 31901298

[B63] Liu Y. Liu Z. Tang H. Shen Y. Gong Z. Xie N. (2019). The N(6)-methyladenosine (m(6)A)-forming enzyme METTL3 facilitates M1 macrophage polarization through the methylation of STAT1 mRNA. Am. J. Physiol. Cell Physiol. 317 (4), C762-C775–C775. 10.1152/ajpcell.00212.2019 31365297

[B64] Maron B. A. Abman S. H. Elliott C. G. Frantz R. P. Hopper R. K. Horn E. M. (2021). Pulmonary arterial hypertension: diagnosis, treatment, and novel advances. Am. J. Respir. Crit. Care Med. 203 (12), 1472–1487. 10.1164/rccm.202012-4317SO 33861689 PMC8483220

[B65] Maron B. A. Brittain E. L. Choudhary G. Gladwin M. T. (2018). Redefining pulmonary hypertension. Lancet Respir. Med. 6 (3), 168–170. 10.1016/S2213-2600(17)30498-8 29269004

[B66] Maston L. D. Jones D. T. Giermakowska W. Howard T. A. Cannon J. L. Wang W. (2017). Central role of T helper 17 cells in chronic hypoxia-induced pulmonary hypertension. Am. J. Physiol. Lung Cell Mol. Physiol. 312 (5), L609-L624–L624. 10.1152/ajplung.00531.2016 28213473 PMC5451600

[B67] Mathiyalagan P. Adamiak M. Mayourian J. Sassi Y. Liang Y. Agarwal N. (2019). FTO-dependent N(6)-methyladenosine regulates cardiac function during remodeling and repair. Circulation 139 (4), 518–532. 10.1161/CIRCULATIONAHA.118.033794 29997116 PMC6400591

[B68] Mauer J. Luo X. Blanjoie A. Jiao X. Grozhik A. V. Patil D. P. (2017). Reversible methylation of m(6)A(m) in the 5' cap controls mRNA stability. Nature 541 (7637), 371–375. 10.1038/nature21022 28002401 PMC5513158

[B69] Mouton A. J. DeLeon-Pennell K. Y. Rivera Gonzalez O. J. Flynn E. R. Freeman T. C. Saucerman J. J. (2018). Mapping macrophage polarization over the myocardial infarction time continuum. Basic Res. Cardiol. 113 (4), 26. 10.1007/s00395-018-0686-x 29868933 PMC5986831

[B70] Mu X. Shi W. Xu Y. Xu C. Zhao T. Geng B. (2018). Tumor-derived lactate induces M2 macrophage polarization via the activation of the ERK/STAT3 signaling pathway in breast cancer. Cell Cycle 17 (4), 428–438. 10.1080/15384101.2018.1444305 29468929 PMC5927648

[B71] Norton C. E. Sheak J. R. Yan S. Weise-Cross L. Jernigan N. L. Walker B. R. (2020). Augmented pulmonary vasoconstrictor reactivity after chronic hypoxia requires src kinase and epidermal growth factor receptor signaling. Am. J. Respir. Cell Mol. Biol. 62 (1), 61–73. 10.1165/rcmb.2018-0106OC 31264901 PMC6938133

[B72] Patil D. P. Chen C. K. Pickering B. F. Chow A. Jackson C. Guttman M. (2016). m(6)A RNA methylation promotes XIST-mediated transcriptional repression. Nature 537 (7620), 369–373. 10.1038/nature19342 27602518 PMC5509218

[B73] Pendleton K. E. Chen B. Liu K. Hunter O. V. Xie Y. Tu B. P. (2017). The U6 snRNA m(6)A methyltransferase METTL16 regulates SAM synthetase intron retention. Cell 169 (5), 824–835. 10.1016/j.cell.2017.05.003 28525753 PMC5502809

[B74] Perros F. Cohen-Kaminsky S. Gambaryan N. Girerd B. Raymond N. Klingelschmitt I. (2013). Cytotoxic cells and granulysin in pulmonary arterial hypertension and pulmonary veno-occlusive disease. Am. J. Respir. Crit. Care Med. 187 (2), 189–196. 10.1164/rccm.201208-1364OC 23220918

[B75] Ping X. L. Sun B. F. Wang L. Xiao W. Yang X. Wang W. J. (2014). Mammalian WTAP is a regulatory subunit of the RNA N6-methyladenosine methyltransferase. Cell Res. 24 (2), 177–189. 10.1038/cr.2014.3 24407421 PMC3915904

[B76] Pucino V. Certo M. Bulusu V. Cucchi D. Goldmann K. Pontarini E. (2019). Lactate buildup at the site of chronic inflammation promotes disease by inducing CD4(+) T cell metabolic rewiring. Cell Metab. 30 (6), 1055–1074. 10.1016/j.cmet.2019.10.004 31708446 PMC6899510

[B77] Qin Y. Qiao Y. Li L. Luo E. Wang D. Yao Y. (2021). The m(6)A methyltransferase METTL3 promotes hypoxic pulmonary arterial hypertension. Life Sci. 274, 119366. 10.1016/j.lfs.2021.119366 33741419

[B78] Qing Y. Dong L. Gao L. Li C. Li Y. Han L. (2021). R-2-hydroxyglutarate attenuates aerobic glycolysis in leukemia by targeting the FTO/m(6)A/PFKP/LDHB axis. Mol. Cell 81 (5), 922–939.e9. 10.1016/j.molcel.2020.12.026 33434505 PMC7935770

[B79] Qu L. H. Luo W. J. Yan Z. G. Liu W. P. (2022). FAM171B as a novel biomarker mediates tissue immune microenvironment in pulmonary arterial hypertension. Mediat. Inflamm. 2022, 1878766. 10.1155/2022/1878766 PMC9553458 36248192

[B80] Rhodes C. J. Batai K. Bleda M. Haimel M. Southgate L. Germain M. (2019). Genetic determinants of risk in pulmonary arterial hypertension: international genome-wide association studies and meta-analysis. Lancet Respir. Med. 7 (3), 227–238. 10.1016/S2213-2600(18)30409-0 30527956 PMC6391516

[B81] Ries R. J. Zaccara S. Klein P. Olarerin-George A. Namkoong S. Pickering B. F. (2019). m(6)A enhances the phase separation potential of mRNA. Nature 571 (7765), 424–428. 10.1038/s41586-019-1374-1 31292544 PMC6662915

[B82] Roundtree I. A. Luo G. Z. Zhang Z. Wang X. Zhou T. Cui Y. (2017). YTHDC1 mediates nuclear export of N(6)-methyladenosine methylated mRNAs. Elife 6, e31311. 10.7554/eLife.31311 28984244 PMC5648532

[B83] Saygin D. Highland K. B. Farha S. Park M. Sharp J. Roach E. C. (2017). Metabolic and functional evaluation of the heart and lungs in pulmonary hypertension by gated 2-[18F]-Fluoro-2-deoxy-D-glucose positron emission tomography. Pulm. Circ. 7 (2), 428–438. 10.1177/2045893217701917 28597761 PMC5467932

[B84] Shafiq M. Jagavelu K. Iqbal H. Yadav P. Chanda D. Verma N. K. (2021). Inhibition of mitogen-activated protein kinase (MAPK)-Activated protein kinase 2 (MK2) is protective in pulmonary hypertension. Hypertension 77 (4), 1248–1259. 10.1161/HYPERTENSIONAHA.120.15229 33641361

[B85] Shi H. Wang X. Lu Z. Zhao B. S. Ma H. Hsu P. J. (2017). YTHDF3 facilitates translation and decay of N(6)-methyladenosine-modified RNA. Cell Res. 27 (3), 315–328. 10.1038/cr.2017.15 28106072 PMC5339834

[B86] Shi H. Zhang X. Weng Y. L. Lu Z. Liu Y. Lu Z. (2018). m(6)A facilitates hippocampus-dependent learning and memory through YTHDF1. Nature 563 (7730), 249–253. 10.1038/s41586-018-0666-1 30401835 PMC6226095

[B87] Shime H. Yabu M. Akazawa T. Kodama K. Matsumoto M. Seya T. (2008). Tumor-secreted lactic acid promotes IL-23/IL-17 proinflammatory pathway. J. Immunol. 180 (11), 7175–7183. 10.4049/jimmunol.180.11.7175 18490716

[B88] Simonneau G. Montani D. Celermajer D. S. Denton C. P. Gatzoulis M. A. Krowka M. (2019). Haemodynamic definitions and updated clinical classification of pulmonary hypertension. Eur. Respir. J. 53 (1), 1801913. 10.1183/13993003.01913-2018 30545968 PMC6351336

[B89] Souto-Carneiro M. M. Klika K. D. Abreu M. T. Meyer A. P. Saffrich R. Sandhoff R. (2020). Effect of increased lactate dehydrogenase A activity and aerobic glycolysis on the proinflammatory profile of autoimmune CD8+ T cells in rheumatoid arthritis. Arthritis Rheumatol. 72 (12), 2050–2064. 10.1002/art.41420 32602217

[B90] Steiner M. K. Syrkina O. L. Kolliputi N. Mark E. J. Hales C. A. Waxman A. B. (2009). Interleukin-6 overexpression induces pulmonary hypertension. Circ. Res. 104 (2), 236–244. 10.1161/CIRCRESAHA.108.182014 19074475 PMC5482545

[B91] Stillman B. (2018). Histone modifications: insights into their influence on gene expression. Cell 175 (1), 6–9. 10.1016/j.cell.2018.08.032 30217360

[B92] Su R. Dong L. Li C. Nachtergaele S. Wunderlich M. Qing Y. (2018). R-2HG exhibits anti-tumor activity by targeting FTO/m(6)A/MYC/CEBPA signaling. Cell 172 (1-2), 90–105. 10.1016/j.cell.2017.11.031 29249359 PMC5766423

[B93] Sun S. Xu X. Liang L. Wang X. Bai X. Zhu L. (2021). Lactic acid-producing probiotic *Saccharomyces cerevisiae* attenuates ulcerative colitis via suppressing macrophage pyroptosis and modulating gut microbiota. Front. Immunol. 12, 777665. 10.3389/fimmu.2021.777665 34899735 PMC8652295

[B94] Tamosiuniene R. Tian W. Dhillon G. Wang L. Sung Y. K. Gera L. (2011). Regulatory T cells limit vascular endothelial injury and prevent pulmonary hypertension. Circ. Res. 109 (8), 867–879. 10.1161/CIRCRESAHA.110.236927 21868697 PMC3204361

[B95] Tang C. Klukovich R. Peng H. Wang Z. Yu T. Zhang Y. (2018a). ALKBH5-dependent m6A demethylation controls splicing and stability of long 3'-UTR mRNAs in male germ cells. Proc. Natl. Acad. Sci. U. S. A. 115 (2), E325-E333–E333. 10.1073/pnas.1717794115 29279410 PMC5777073

[B96] Tang C. Luo Y. Li S. Huang B. Xu S. Li L. (2021). Characteristics of inflammation process in monocrotaline-induced pulmonary arterial hypertension in rats. Biomed. Pharmacother. 133, 111081. 10.1016/j.biopha.2020.111081 33378977

[B97] Tang H. Babicheva A. McDermott K. M. Gu Y. Ayon R. J. Song S. (2018b). Endothelial HIF-2α contributes to severe pulmonary hypertension due to endothelial-to-mesenchymal transition. Am. J. Physiol. Lung Cell Mol. Physiol. 314 (2), L256-L275–L275. 10.1152/ajplung.00096.2017 29074488 PMC5866501

[B98] Tian W. Jiang X. Tamosiuniene R. Sung Y. K. Qian J. Dhillon G. (2013). Blocking macrophage leukotriene b4 prevents endothelial injury and reverses pulmonary hypertension. Sci. Transl. Med. 5 (200), 200ra117. 10.1126/scitranslmed.3006674 PMC4016764 23986401

[B99] Varner E. L. Trefely S. Bartee D. von Krusenstiern E. Izzo L. Bekeova C. (2020). Quantification of lactoyl-CoA (lactyl-CoA) by liquid chromatography mass spectrometry in mammalian cells and tissues. Open Biol. 10 (9), 200187. 10.1098/rsob.200187 32961073 PMC7536085

[B100] Vasconcelos E. S. J. Simao D. Terrasso A. P. Silva M. M. Brito C. Isidro I. A. (2020). Unveiling dynamic metabolic signatures in human induced pluripotent and neural stem cells. PLoS Comput. Biol. 16 (4), e1007780. 10.1371/journal.pcbi.1007780 32298259 PMC7188302

[B101] Vu L. P. Pickering B. F. Cheng Y. Zaccara S. Nguyen D. Minuesa G. (2017). The N(6)-methyladenosine (m(6)A)-forming enzyme METTL3 controls myeloid differentiation of normal hematopoietic and leukemia cells. Nat. Med. 23 (11), 1369–1376. 10.1038/nm.4416 28920958 PMC5677536

[B102] Wang J. Yan S. Lu H. Wang S. Xu D. (2019). METTL3 attenuates LPS-induced inflammatory response in macrophages via NF-κB signaling pathway. Mediat. Inflamm. 2019, 3120391. 10.1155/2019/3120391 PMC6854952 31772500

[B103] Wang K. Jiang L. Zhang Y. Chen C. (2020). Progression of thyroid carcinoma is promoted by the m6A methyltransferase METTL3 through regulating m(6)A methylation on TCF1. Onco Targets Ther. 13, 1605–1612. 10.2147/OTT.S234751 32158230 PMC7044742

[B104] Wang R. R. Yuan T. Y. Chen D. Chen Y. C. Sun S. C. Wang S. B. (2022a). Dan-shen-yin granules prevent hypoxia-induced pulmonary hypertension *via* STAT3/HIF-1α/VEGF and FAK/AKT signaling pathways. Front. Pharmacol. 13, 844400. 10.3389/fphar.2022.844400 35479305 PMC9035666

[B105] Wang X. Li Q. He S. Bai J. Ma C. Zhang L. (2022b). LncRNA FENDRR with m6A RNA methylation regulates hypoxia-induced pulmonary artery endothelial cell pyroptosis by mediating DRP1 DNA methylation. Mol. Med. 28 (1), 126. 10.1186/s10020-022-00551-z 36284300 PMC9594874

[B106] Wang X. Zhao B. S. Roundtree I. A. Lu Z. Han D. Ma H. (2015). N(6)-methyladenosine modulates messenger RNA translation efficiency. Cell 161 (6), 1388–1399. 10.1016/j.cell.2015.05.014 26046440 PMC4825696

[B107] Watanabe R. Hilhorst M. Zhang H. Zeisbrich M. Berry G. J. Wallis B. B. (2018). Glucose metabolism controls disease-specific signatures of macrophage effector functions. JCI Insight 3 (20), e123047. 10.1172/jci.insight.123047 30333306 PMC6237479

[B108] Watson M. J. Vignali P. D. A. Mullett S. J. Overacre-Delgoffe A. E. Peralta R. M. Grebinoski S. (2021). Metabolic support of tumour-infiltrating regulatory T cells by lactic acid. Nature 591 (7851), 645–651. 10.1038/s41586-020-03045-2 33589820 PMC7990682

[B109] Wen J. Cheng S. Zhang Y. Wang R. Xu J. Ling Z. (2021). Lactate anions participate in T cell cytokine production and function. Sci. China Life Sci. 64 (11), 1895–1905. 10.1007/s11427-020-1887-7 33580429

[B110] Wen J. Lv R. Ma H. Shen H. He C. Wang J. (2018). Zc3h13 regulates nuclear RNA m(6)A methylation and mouse embryonic stem cell self-renewal. Mol. Cell 69 (6), 1028–1038. 10.1016/j.molcel.2018.02.015 29547716 PMC5858226

[B111] Wu B. Su S. Patil D. P. Liu H. Gan J. Jaffrey S. R. (2018). Molecular basis for the specific and multivariant recognitions of RNA substrates by human hnRNP A2/B1. Nat. Commun. 9 (1), 420. 10.1038/s41467-017-02770-z 29379020 PMC5789076

[B112] Xiao Y. Peng H. Hong C. Chen Z. Deng X. Wang A. (2017). PDGF promotes the Warburg effect in pulmonary arterial smooth muscle cells via activation of the PI3K/AKT/mTOR/HIF-1α signaling pathway. Cell Physiol. Biochem. 42 (4), 1603–1613. 10.1159/000479401 28738389

[B113] Xiong J. He J. Zhu J. Pan J. Liao W. Ye H. (2022). Lactylation-driven METTL3-mediated RNA m(6)A modification promotes immunosuppression of tumor-infiltrating myeloid cells. Mol. Cell 82 (9), 1660–1677.e10. 10.1016/j.molcel.2022.02.033 35320754

[B114] Xu D. Li Y. Zhang B. Wang Y. Liu Y. Luo Y. (2016). Resveratrol alleviate hypoxic pulmonary hypertension via anti-inflammation and anti-oxidant pathways in rats. Int. J. Med. Sci. 13 (12), 942–954. 10.7150/ijms.16810 27994500 PMC5165688

[B115] Xu S. Xu X. Zhang Z. Yan L. Zhang L. Du L. (2021a). The role of RNA m(6)A methylation in the regulation of postnatal hypoxia-induced pulmonary hypertension. Respir. Res. 22 (1), 121. 10.1186/s12931-021-01728-6 33902609 PMC8074209

[B116] Xu Z. Ruan J. Pan L. Chen C. (2021b). Candidate genes identified in systemic sclerosis-related pulmonary arterial hypertension were associated with immunity, inflammation, and cytokines. Cardiovasc Ther. 2021, 6651009. 10.1155/2021/6651009 33680092 PMC7906811

[B117] Yang K. Fan M. Wang X. Xu J. Wang Y. Tu F. (2022). Lactate promotes macrophage HMGB1 lactylation, acetylation, and exosomal release in polymicrobial sepsis. Cell Death Differ. 29 (1), 133–146. 10.1038/s41418-021-00841-9 34363018 PMC8738735

[B118] Yang W. Wang P. Cao P. Wang S. Yang Y. Su H. (2021). Hypoxic *in vitro* culture reduces histone lactylation and impairs pre-implantation embryonic development in mice. Epigenetics Chromatin 14 (1), 57. 10.1186/s13072-021-00431-6 34930415 PMC8691063

[B119] Yao M. Z. Ge X. Y. Liu T. Huang N. Liu H. Chen Y. (2020). MEIS1 regulated proliferation and migration of pulmonary artery smooth muscle cells in hypoxia-induced pulmonary hypertension. Life Sci. 255, 117822. 10.1016/j.lfs.2020.117822 32450174

[B120] Yu J. Chai P. Xie M. Ge S. Ruan J. Fan X. (2021). Histone lactylation drives oncogenesis by facilitating m(6)A reader protein YTHDF2 expression in ocular melanoma. Genome Biol. 22 (1), 85. 10.1186/s13059-021-02308-z 33726814 PMC7962360

[B121] Zaccara S. Jaffrey S. R. (2020). A unified model for the function of YTHDF proteins in regulating m(6)a-modified mRNA. Cell 181 (7), 1582–1595. 10.1016/j.cell.2020.05.012 32492408 PMC7508256

[B122] Zeng Y. Huang T. Zuo W. Wang D. Xie Y. Wang X. (2021). Integrated analysis of m(6)A mRNA methylation in rats with monocrotaline-induced pulmonary arterial hypertension. Aging (Albany NY) 13 (14), 18238–18256. 10.18632/aging.203230 34310344 PMC8351682

[B123] Zhang D. Tang Z. Huang H. Zhou G. Cui C. Weng Y. (2019). Metabolic regulation of gene expression by histone lactylation. Nature 574 (7779), 575–580. 10.1038/s41586-019-1678-1 31645732 PMC6818755

[B124] Zhang N. Jiang N. Yu L. Guan T. Sang X. Feng Y. (2021). Protein lactylation critically regulates energy metabolism in the Protozoan parasite trypanosoma brucei. Front. Cell Dev. Biol. 9, 719720. 10.3389/fcell.2021.719720 34722503 PMC8551762

[B125] Zhang Q. Chen Y. Wang Q. Wang Y. Feng W. Chai L. (2023). HMGB1-induced activation of ER stress contributes to pulmonary artery hypertension *in vitro* and *in vivo* . Respir. Res. 24 (1), 149. 10.1186/s12931-023-02454-x 37268944 PMC10236651

[B126] Zhang S. Liu X. Ge L. L. Li K. Sun Y. Wang F. (2020). Mesenchymal stromal cell-derived exosomes improve pulmonary hypertension through inhibition of pulmonary vascular remodeling. Respir. Res. 21 (1), 71. 10.1186/s12931-020-1331-4 32192495 PMC7082982

[B127] Zhang S. Zhao B. S. Zhou A. Lin K. Zheng S. Lu Z. (2017). m(6)A demethylase ALKBH5 maintains tumorigenicity of glioblastoma stem-like cells by sustaining FOXM1 expression and cell proliferation program. Cancer Cell 31 (4), 591–606. 10.1016/j.ccell.2017.02.013 28344040 PMC5427719

[B128] Zhang Y. Hernandez M. Gower J. Winicki N. Morataya X. Alvarez S. (2022). JAGGED-NOTCH3 signaling in vascular remodeling in pulmonary arterial hypertension. Sci. Transl. Med. 14 (643), eabl5471. 10.1126/scitranslmed.abl5471 35507674

[B129] Zhao Y. Peng J. Lu C. Hsin M. Mura M. Wu L. (2014). Metabolomic heterogeneity of pulmonary arterial hypertension. PLoS One 9 (2), e88727. 10.1371/journal.pone.0088727 24533144 PMC3923046

[B130] Zheng H. Hua J. Li H. He W. Chen X. Ji Y. (2022). Comprehensive analysis of the expression of N6-methyladenosine RNA methylation regulators in pulmonary artery hypertension. Front. Genet. 13, 974740. 10.3389/fgene.2022.974740 36171892 PMC9510777

[B131] Zhou X. L. Huang F. J. Li Y. Huang H. Wu Q. C. (2021). SEDT2/METTL14-mediated m6A methylation awakening contributes to hypoxia-induced pulmonary arterial hypertension in mice. Aging (Albany NY) 13 (5), 7538–7548. 10.18632/aging.202616 33658391 PMC7993666

[B132] Zhu B. Gong Y. Shen L. Li J. Han J. Song B. (2020). Total Panax notoginseng saponin inhibits vascular smooth muscle cell proliferation and migration and intimal hyperplasia by regulating WTAP/p16 signals via m(6)A modulation. Biomed. Pharmacother. 124, 109935. 10.1016/j.biopha.2020.109935 31986407

[B133] Zhu F. Yang T. Yao M. Shen T. Fang C. (2021a). HNRNPA2B1, as a m(6)A reader, promotes tumorigenesis and metastasis of oral squamous cell carcinoma. Front. Oncol. 11, 716921. 10.3389/fonc.2021.716921 34631545 PMC8494978

[B134] Zhu X. Yang H. Zhang M. Wu X. Jiang L. Liu X. (2021b). YTHDC1-mediated VPS25 regulates cell cycle by targeting JAK-STAT signaling in human glioma cells. Cancer Cell Int. 21 (1), 645. 10.1186/s12935-021-02304-0 34863175 PMC8642909

